# Multifunctional Supramolecular Hybrid Materials Constructed from Hierarchical Self-Ordering of In Situ Generated Metal-Organic Framework (MOF) Nanoparticles

**DOI:** 10.1002/adma.201501448

**Published:** 2015-06-25

**Authors:** Abhijeet K Chaudhari, Intaek Han, Jin-Chong Tan

**Affiliations:** Department of Engineering Science, University of OxfordParks Road, Oxford, OX1 3PJ, UK E-mail: jin-chong.tan@eng.ox.ac.uk; Materials Research Center, Samsung Advanced Institute of Technology (SAIT), Samsung ElectronicsSuwon, 443-803, Republic of Korea

**Keywords:** hierarchical self-assembly, metal-organic frameworks, sol–gel, stimuli-responsive materials, supramolecular materials

Major advances in supramolecular science[1] have triggered a new wave of discoveries of next-generation functional materials, especially stimuli-responsive soft matter,[2] which could afford multiple functions applicable to emergent technologies. It is envisioned that the unique combination of self-organized supramolecular systems,[3] self-healing[4] and thermo-reversibility,[5] and on-demand sol–gel transformation[6] makes stimuli-responsive materials not only extremely versatile, but also straightforward to process into novel multifunctional devices.[7] Yet, the precise control over its microstructural alignment and detailed understanding of complex organization, constituting a self-assembly,[1] remain some of the biggest challenges facing supramolecular materials science today. One of the best examples featuring self-organization of lamellar hierarchical growth lies in the naturally occurring protein assembly, known as collagen,[8] whose highly aligned fibrous microstructure not only dictates its biomechanical stability, but also underpins numerous biostimuli functions.[9] To this end, exploration of synthetic routes mimicking the self-directed molecular assemblies evidenced in nature has attracted considerable interests, further broadening the scope of applications linked to novel functional materials and soft matter. For example, oriented growth of supramolecular assemblies allows switchable or tunable material properties, which have utility in emerging areas ranging from microfluidics and molecular filtration, to nanowires and microelectronics devices.[7] Recent studies have also focused on the cooperative effects arising from nanoparticle–polymer pairs (or block copolymers) to create energetically favorable multicomponent systems, incorporating complex patterned growth of bespoke nanomaterials.[10]

In this study, we report a rare type of supramolecular hybrid material built from self-organization of nano-metal-organic framework (NMOF) particles, which are accommodated within highly aligned fiber network scaffolding. Metal-organic framework (MOF)[11] is a rapidly expanding class of crystalline nano­porous materials, whose 3D framework consists of ordered units of metal ions or clusters bridged by organic linkages. MOFs offer rich chemical functionalities combined with vast structural versatility.[12] The high uniformity and 3D microporous architecture of MOF crystals could provide the unique platform for symbiotic effects, yielding orthogonal interactions[13] central to achieve supramolecular self-assembly. It is worth noting that, although metal-organic gels (MOGs)[14] are a very similar type of self-assembled hybrid compound, they comprise randomly cross-linked metal ions by organic linkers (without forming an ordered framework), subsequently trapping solvent molecules to form a more conventional gel network material.

The microporous material designated as HKUST-1[15] represents one of the most intensely studied MOFs today due to its wide-ranging potential applications. Typically, it can be obtained via solvothermal reactions between Cu(II) and BTC^3−^ (1,3,5-benzene tricarboxylic acid), yielding crystalline HKUST-1 [Cu_3_(BTC)_2_] as the most thermodynamically favorable product.[16] In fact, HKUST-1 is renowned for its ease of synthesis using different solvents, temperatures, or bases,[17] in addition to membranes, hollow capsules, and superstructures derived from it.[18] Herein, we demonstrate that, by employing a high concentration of standard reactants of HKUST-1 in a relatively small quantity of solvents, yields previously unreported gel-like hybrid materials that exhibit counterintuitive chemico-physical properties.

In the present work, we discovered that room-temperature reaction between Cu(NO_3_)_2_ solution and deprotonated BTC, using triethylamine base (NEt_3_), yields facile formation of an unconventional gel-like supramolecular self-assembly. Interestingly, the aforementioned reactions can be accomplished in both polar-protic and polar-aprotic solvents, resulting in an entirely new system of MOF-based supramolecular hybrid materials (see Figure S1 in the Supporting Information), which we termed: **G⊃ACN**, **G⊃DMF**, **G⊃DMSO**, **G⊃ETH**, and **G⊃MEH** (where G denotes gel obtained using solvent: ACN: acetonitrile; DMF: *N*,*N*-dimethyl formamide; DMSO: dimethyl sulfoxide; ETH: ethanol; MEH: methanol; see Figure S2 and Table S1 in the Supporting Information). The formation mechanism was investigated utilizing different solvents, which allowed us to study the rich morphological and structural diversity of these novel hybrid materials. We found that the use of different solvents yields hybrid materials that exhibit distinct structural, mechanical, chemical, and electrical properties. Noteworthy, sol–gel transitions occur only in the case of **G⊃DMSO**, while viscoelastic phase conversion (from soft to rigid network) is evident only in **G⊃ACN**. Moreover, detailed characterization of physico-mechanical properties has enabled us to pinpoint structural dependencies of uncommon functions. The abovementioned are key highlights of the current study which are explored in detail in this work.

Application of DMSO as a solvent for making solutions of Cu(NO_3_)_2_ and BTC produces a hybrid gel compound featuring unique properties, as depicted in **Figure** **1**. Although the reaction of BTC and Cu(II) in DMSO solvent has previously been reported,[19] in our approach, the addition of triethylamine (NEt_3_) triggers an unexpected auxiliary effect leading to the formation of a new supramolecular material. Remarkably, this hybrid gel compound, **G⊃DMSO**, exhibits multiple responses against thermal, mechanical, and chemical stimuli (Figure 1a). First, it was found that subsequent alternate additions of 1 equivalent of BTC and/or 1.5 equivalent of Cu(II) solution cause the hybrid assembly to undergo a rapid phase transformation, i.e., switching from gel to sol, and vice versa. This observation confirms that the hybrid gel can be broken down by dissolution, but subsequently reassembled upon the addition of cationic Cu(II) or anionic BTC^3−^, such that chemical triggering accounts for the reversible nature of sol–gel transformation observed. Nevertheless, as this process is repeated with successive cycles of additions of BTC and/or Cu(II) solutions, an increasingly longer time was required to recover the gel form (Table S2, Supporting Information). Second, mechanical stresses invoked by shaking could disrupt the structural integrity of the **G⊃DMSO** hybrid assembly, causing the gel to collapse into a viscous sol (Figure 1a). This gel phase, however, can be regained either by subjecting the sol to sonication for ≈1–2 min, or simply by leaving it uninterrupted for ≈10–15 min. The gel was found to be extremely sensitive to application of external forces such that phase recovery (sol to gel) takes longer to occur when gel is mechanically agitated for a longer period of time. Third, further to chemical and mechanical responses, this particular hybrid gel also demonstrates thermo-responsive behavior; for instance, the sol of **G⊃DMSO** can be transformed back into a gel when heated at 80 °C for just ≈1–2 min.

**Figure 1:**
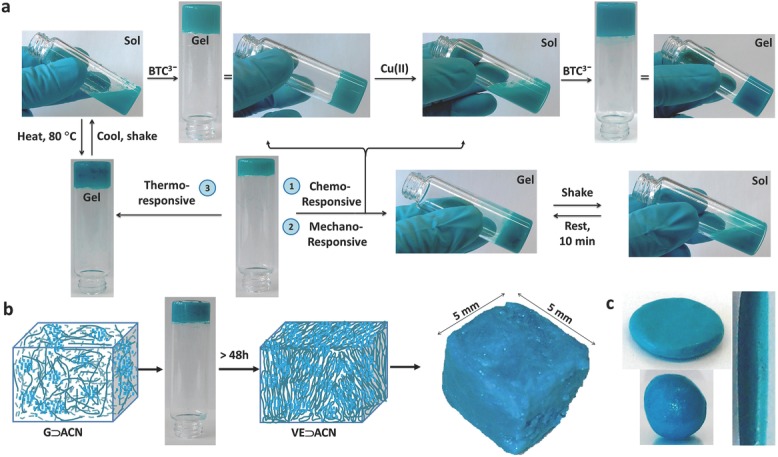
a) Multistimuli chemo–mechano–thermo-responsive behavior enabled via sol–gel transformation of G⊃DMSO. b) Schematic illustration showing phase transformation of G⊃ACN, after ≈48 h, forming the viscoelastic material, termed VE⊃ACN. Schematics: fibrous strands (dark green) represent the 3D network scaffolding, which is integrated with HKUST-1 NMOF particles (light blue). Right: a cuboid measuring 5 × 5 × 5 mm^3^ molded from a monolith of VE⊃ACN. c) Examples of several 3D objects made from VE⊃ACN.

We identified another striking phase change phenomenon conferred by the acetonitrile solvent (ACN), wherein the corresponding hybrid gel synthesized is designated as **G⊃ACN**. Initial observation of **G⊃ACN** gels transforming into a semi-solid-like material has prompted us to study this effect in detail. We established that by allowing an **G⊃ACN** sample (sealed in a vial) unperturbed for more than 48 h would eventually transform the gel phase into a viscoelastic (VE) material (Figure 1b,c), which we termed **VE⊃ACN** (detailed viscoelastic mechanical behavior presented in later sections). Its two major characteristics are: (1) if gentle mechanical stress is imposed onto **G⊃ACN** in the gel state (for instance, by shaking sample vial), this causes the fibrous gel scaffolding to break down to yield a sol; and (2) more vigorous mechanical agitation of the reactant mixture ultimately produces only precipitation, prohibiting any further gelation from occurring. Above findings support the notion that gel to viscoelastic phase change can be accomplished, only if the underlying gel scaffolding (i.e. building skeleton) is kept stress-free and maintained stable with encapsulated solvent molecules. In light of this, the foundation for constructing a rigid fiber network stems from the gel itself, acting as the 3D scaffold; but without which, phase change into the viscoelastic material cannot occur. An attractive physical property of the **VE⊃ACN** material lies in its shape-persistent capacity; it is mechanically resilient enough to be dissected, deformed, or molded into a range of 3D shapes, a few examples of which are illustrated in Figure 1b,c.

**Figure** **2** shows microstructural characteristics of the supramolecular hybrid gel materials obtained from scanning electron microscopy (SEM), revealing exquisite fine-scale morphologies that correlate to their chemical structures and physical properties (Figure 2 and Figures S3–S8, Supporting Information). Comparing Figure 2a to b, we recognize that the viscoelastic material (**VE⊃ACN**) consists of fine fibrous filaments hierarchically packed within fiber bundles, which are reminiscent of fibrils present in structural biological materials.[8,9] Such a microstructure, however, is absent in the initial gel material (**G⊃ACN**) prior to its viscoelastic conversion. It can be seen that images of the **G⊃DMSO** system that displays multistimuli response reveal relatively thicker rods of gel-like blocks; two examples of which are shown in Figure 2c,d with dimensions of ≈200 × 50 μm. In contrast, the remaining gel samples (Figure 2e–j) feature appreciably smaller bundles of microscopic fibers, growing unidirectionally in the form of strips, whose lengths may extend from several micrometers up to hundreds of micrometers. It is worth noting that recent literature on MOF-based aerogels,[20] xerogels,[21] and metal-organic gels (MOGs)[14c,^22^] exhibits substantially different fine-scale microstructures, compared with the distinctive fiber microarchitectures we reported here.

**Figure 2:**
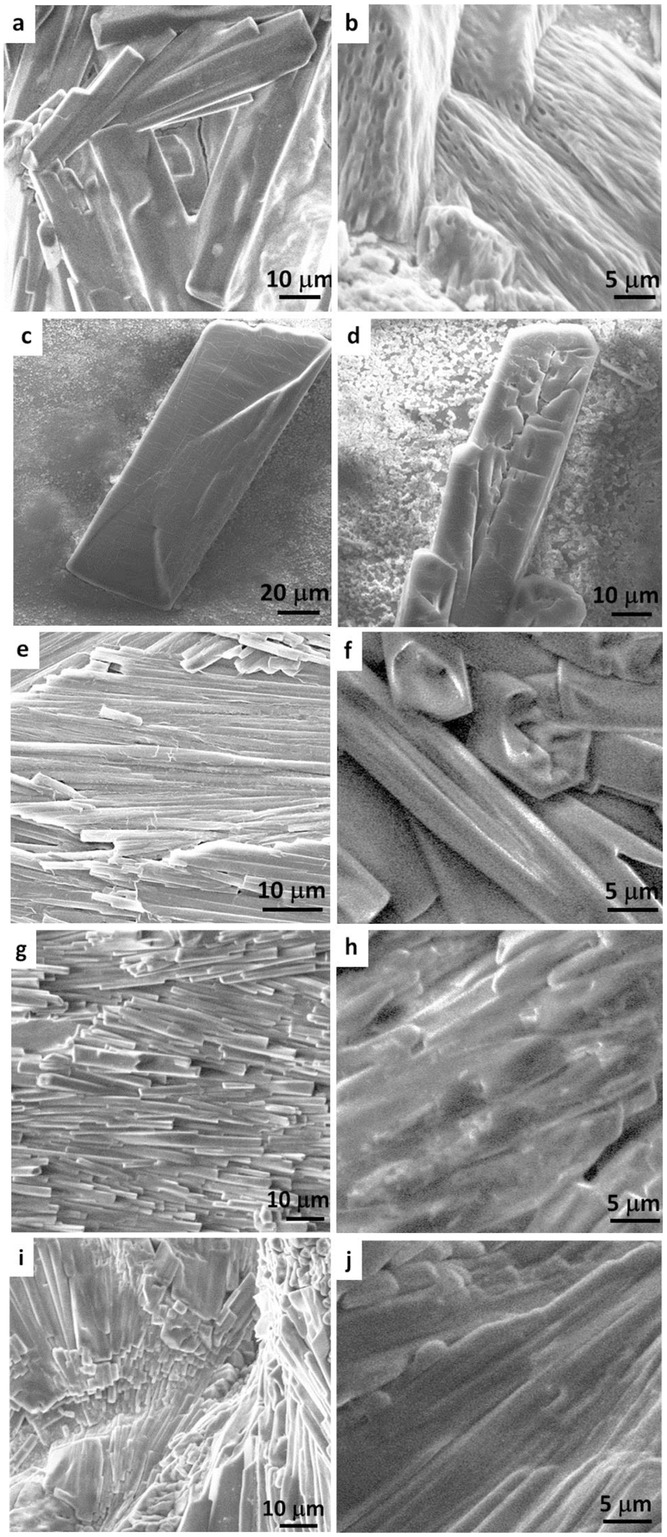
SEM images of hierarchically self-assembled gel microstructures (dried samples) obtained from synthesis employing different solvents. a) G⊃ACN and b) its corresponding viscoelastic material VE⊃ACN, c,d) G⊃DMSO, e,f) G⊃MEH, g,h) G⊃DMF, i,j) G⊃ETH.

Given the identical chemical precursors employed in all syntheses, apart from the choice of solvent, it is intriguing that sol–gel transformation was identified only in the case of **G⊃DMSO**, whereas viscoelastic conversion evidenced only in the case of **G⊃ACN**. This result underscores the critical roles played by the specific solvent molecules (Figure S2, Supporting Information), which in turn affect the microstructures of the resultant fiber network assembly (Figure 2) and henceforth its physico-chemical characteristics. Electron donor atoms, viz., O, N, S, which are present in certain solvent molecules enhance interaction with Cu(II) centres, and may extend the structural connectivity by means of hydrogen bonding. Especially in the DMSO solvent, for example, we propose that soft S and hard O donor atoms (at opposing ends of molecule) facilitate switchable connections for weak bond making-breaking process; this mechanism could accommodate supramolecular association–dissociation processes, thus triggering sol–gel conversion of **G⊃DMSO** (Figure 1). Moreover, because S and O readily interact with soft and hard electrophiles, DMSO forms strong hydrogen bonding (Kamlet–Taft parameters: *α* = 0.00, *β* = 0.76)[23] with BTC (Figures S9 and S10, Supporting Information).[24] Of the systems we studied, **G⊃DMSO** requires the longest gelation time (Table S1, Supporting Information), suggesting that the reaction responsible for fiber formation is occurring at a relatively slower pace, due to effects of solvent interaction that may cage BTC^3−^ linkers approaching the Cu(II) cations. Although O donor is also present in other solvent molecules, we found that monodentate ligation property is inadequate to trigger microstructural reintegration fundamental to reversible sol–gel conversions. Turning to **G⊃ACN**, the linear molecular structure of acetonitrile (ACN) means that, not only its associated geometrical constraints should be weaker, but also it offers opportunity for coordination to the Cu(II) centers to further stabilize the overall supramolecular network assembly. Together, it is projected that such auxiliary effects, over time, perpetuate transformation of gel into the viscoelastic material, which constitutes fine-scale fibrous architecture specific to **VE⊃ACN** (Figure 2b and Figure S3, Supporting Information).

By virtue of the rapid formation of **G⊃MEH** gels from methanol (≈2 min), we probed this material to gain insights into the basic structural development mechanisms. During the reaction, we observed intense color emerging from nanoparticles embedded in the fibers as time advances; these nanoparticles can be easily harvested from embedding fibrous matter by washing the gel thrice using excessive methanol (Figure S11, Supporting Information). Bulk amount of nanoparticles collected this way has been examined by powder X-ray diffraction (PXRD, Figure S12, Supporting Information), scanning electron microscopy (SEM, Figure S11, Supporting Information), and atomic force microscopy (AFM, Figure S13, Supporting Information), confirming that they are in fact nanometer-sized MOF (NMOF) particles of phase pure HKUST-1.

We rationalize that the mechanism underpinning the construction of supramolecular MOF hybrid progresses in accordance with **Scheme** **1**a–d, endorsed by experimental observations. At the beginning of the self-assembly process, organic and inorganic basic building blocks coalesce via weak interactions in a nascent network of gel material. This development is subsequently accompanied by stronger coordination bonds forming between Cu(II) and BTC^3−^; despite overcoming weaker molecular interactions, it caused no collapse to the former assembly, therefore yielding HKUST-1 nanoparticles in the fiber network.

**Scheme 1:**
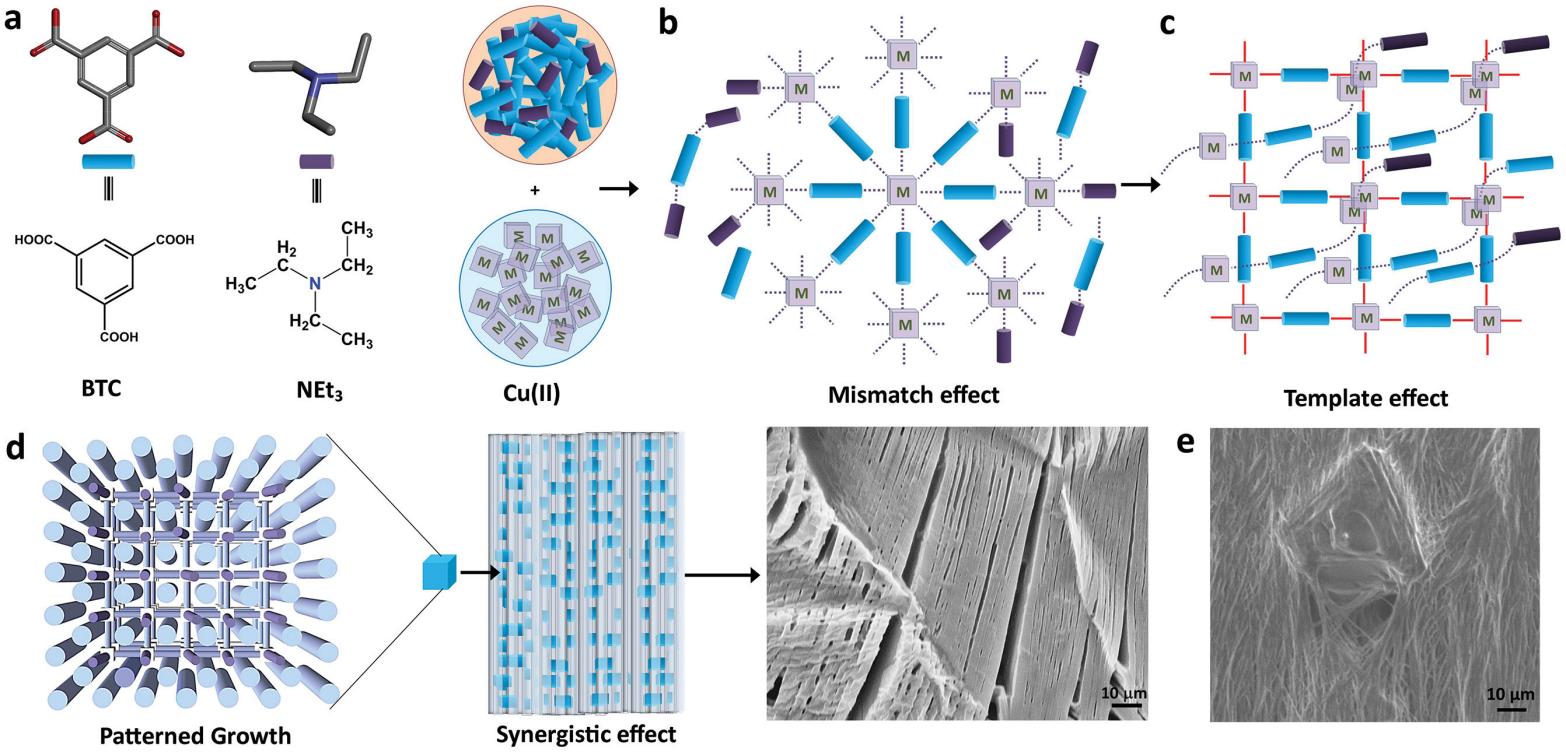
Schematic illustrating the proposed mechanism underpinning hierarchical growth of MOF hybrid network encompassing highly directional fibers. a) Fundamental building blocks react to create b) weak interaction with each other via the mismatch effect. c) Templating effect involving development of coordinating bonds between BTC^3−^ and Cu(II), facilitated by interpenetration of 1D networks bound by weak interactions. d) Patterned growth by means of the synergistic effects between nanoparticles and self-assembled fibrous structure, informed from SEM image of G⊃MEH (Figure S14, Supporting Information) containing nano-HKUST-1 particles integrated in fibers (see Figure 3g). Note: Black dotted lines represent weak intermolecular interactions; red bold lines denote coordination bonding. e) Formation of a defective crystal of HKUST-1 embedded in the fiber network of G⊃DMSO.

Recent reports on interconvertible polymer–metal-organic framework (poly-MOF)[25] hybrid materials and conversion of MOFs into gel-like MOFs[26] are worth describing here for comparison. In the case of poly-MOFs, amorphous polymers bearing coordinating donor atoms (in repeating units) interact with metal ions to form MOF-containing hybrid material. Gel-like MOF material,[26] on the other hand, is thought to form by interconnection of functionalized cross-linking organic linkers present in the MOF. In contrast, the supramolecular assembly of the current series of hybrid materials follows an entirely different mechanism. Distinctively, MOF nanoparticles evolved by overcoming the weakly interacting species present in the nascent assembly, which ultimately yields hierarchical architecture where NMOFs constitute the major crystalline phase of the overall soft matter. Abundance of HKUST-1 NMOF particles and unoccupied metal sites in HKUST-1 crystal lattice offers prospect to other molecules to either coordinate or penetrate the intrinsically hollow MOF channels (resembling templating effects, Scheme 1c). We infer that the threefold rotational symmetry of BTC molecules, paired with coordination to Cu(II) centres, may direct growth of fibrous network in a highly aligned fashion (patterning effect). Moreover, the use of NEt_3_ for deprotonating BTC generates cationic NEt_3_^+^ species; we noted from the literature[27] the propensity of NEt_3_ to coordinate with Cu(II) in the presence of coordinating organic ligands. Such a mechanism, termed the “mismatching molecular effect,”[20b] creates competition between multiple coordinating molecular species alongside NMOF formation due to the resultant synergistic effects (Scheme 1d).

To further understand the growth behavior, in another experiment we have interrogated the effects of uneven dissemination of starting materials on the self-assembly mechanism. A layer of BTC^3−^ solution in DMSO (solution viscosity 3.92 × 10^−2^ Pa s) was first coated on the glass substrate by dip coating (Figure S15, Supporting Information), on the top of which was subsequently introduced several droplets of Cu(II) solution containing DMSO. This approach generates a concentration gradient attributed to the localized mixing of organic and inorganic precursors. Because DMSO is a high-boiling-point solvent, the wet sample of **G⊃DMSO** obtained was left to dry at room temperature; SEM images acquired from the dried sample revealed interesting microstructural attributes elucidating the synergistic self-assembly process, see Scheme 1e. This finding reveals not only that, NMOF particles integrated in nanometer-sized fibers could behave as small seeds (nuclei) that lead to the formation of larger HKUST-1 crystals, but also because of mismatching molecular interactions,[20b] defective crystals featuring screw-like dislocations[28] have evolved on the hybrid gel fiber networks (see also Figures S15 and S16 in the Supporting Information). Here, it is worth emphasizing that normal synthesis of hybrid materials (performed in glass vials with uniform mixing of reactants) will not result in the formation of such defective crystals.

Dynamic rheological experiments were performed to study the structural integrity of the supramolecular hybrid MOFs, when subjected to shear deformation (*γ*) and corresponding shear stress (*τ*). The main results are summarized in **Figure** **3**, where the storage modulus (*G*′) and loss modulus (*G*″) are measures of the recoverable elastic response and dissipative viscous behavior, respectively. It follows that the shear modulus (*G* = *τ*/*γ*), whose magnitude is 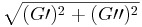, thereby reflects the material's mechanical rigidity, i.e., its structural resistance (stiffness) against distortion caused by a shear deformation. From Figure 3a,b, it can be seen that the storage (*G*′) and loss (*G*″) moduli of the gel samples are distinct from one another, which indicates tunable mechanical properties of this family of hybrid materials based on judicious choice of solvents (Figures S17–S22, Supporting Information). On one hand, higher storage moduli of the order of *G*′ ≈ 15–25 kPa have been determined for **G⊃ACN**, **G⊃DMF**, and **G⊃ETH**; on the other hand, significantly lower magnitudes were found for **G⊃DMSO** (≈5 kPa) and **G⊃MEH** (≈1 kPa), highlighting that an exceedingly low shear modulus is also attainable (Figure S23, Supporting Information).

**Figure 3:**
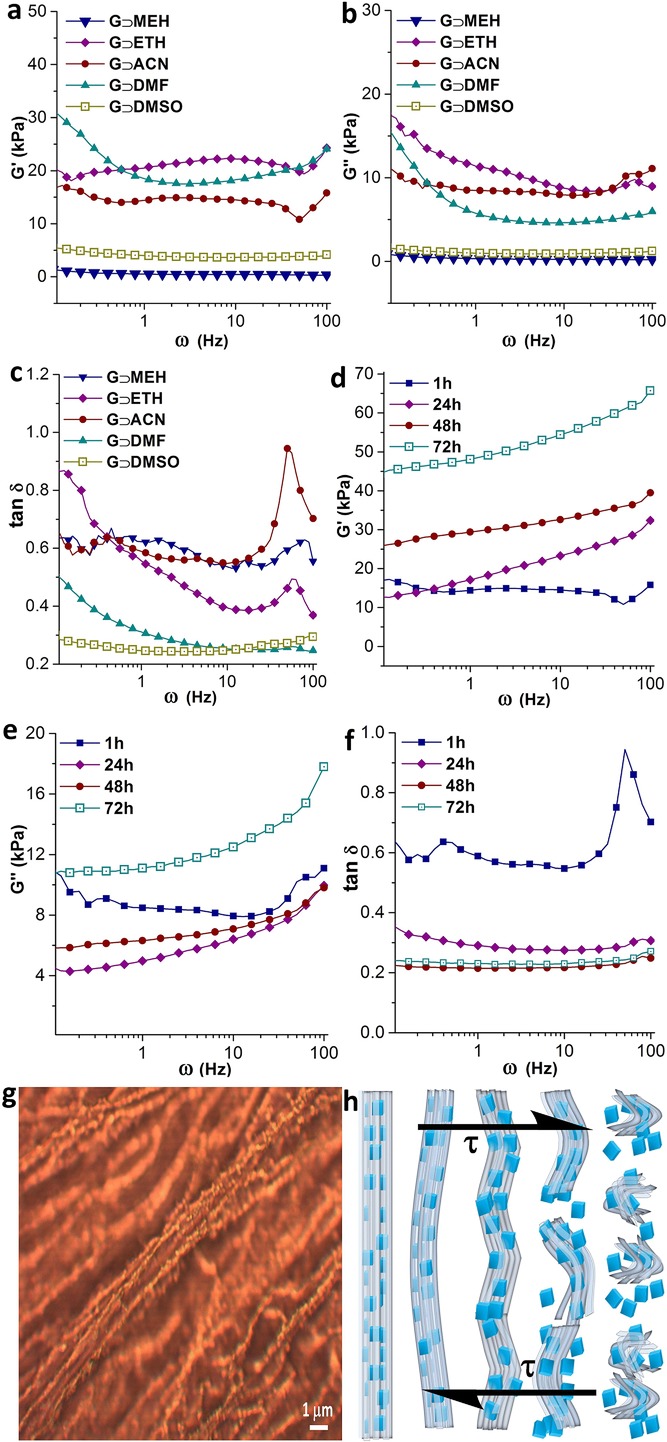
Frequency sweep rheological properties under shear deformation for all hybrid materials as a function of oscillatory angular frequency, *ω* = 0.1–100 Hz: strain = 1%, a) storage moduli *G*′, b) loss moduli *G*″, and c) loss tangent (tan *δ = G*″/*G*′). All samples were tested after 1 h from the point of material synthesis (because gelation time in different solvents varies). Evolution of the mechanical response of G⊃ACN measured at 1, 24, 48, 72 h after material synthesis, each of which acquired using a different fresh sample: d) storage moduli, e) loss moduli, and f) loss tangent data, indicating phase stability of VE⊃ACN beyond ≈48 h. g) Optical microscopy image showing highly ordered HKUST-1 nanoparticles embedded inside the fibers, and h) schematic representing the proposed microstructural changes occurring over phase transition in the hybrid fiber bundles subjected to an opposing pair of shear stresses *τ*.

The loss tangent (tan *δ* = *G*″/*G*′) versus frequency plot in Figure 3c shows characteristic peaks appearing in **G⊃ACN**, **G⊃MEH**, and **G⊃ETH**, corresponding to phase changes associated with higher angular frequencies between ≈50 and 100 Hz. Particularly large deviation was observed in **G⊃ACN**, indicating that its hybrid network is relatively weaker against any shear forces imposed at higher frequencies; conversely, **G⊃DMSO** demonstrates virtually a negligible phase change implying good mechanical stability towards shear deformation. Thixotropic nature[29] of the **G⊃DMSO** sample has been investigated by performing dynamic strain sweep measurements,[30] which confirmed the reversible recovery of the gel phase (within 15 min, over shear strain relaxation from 100% to 0.1% at 0.1 Hz angular frequency, see Figure S24 in the Supporting Information). Herein, we elucidate the shear-induced disintegration of the supramolecular network assembly using the schematic proposed in Figure 3h, in accordance with the observed fibrous architecture integrating HKUST-1 nanoparticles, as exemplified in Figure 3g. Indeed, the thixotropic timescales[29] of the order of minutes have been established in **G⊃DMSO**, for which could be linked to the high concentration of nano-MOF particles affecting the nature of supramolecular hybrid breakdown and its subsequent reconstruction (i.e. recovery upon strain removal).

By monitoring the structural transitions of **G⊃ACN** via rheology, we have established how the conversion into subsequent viscoelastic phase (**VE⊃ACN**) alters the overall network rigidity. Figure 3d,e presents the storage and loss moduli data, corresponding to 1, 24, 48, and 72 h, where dynamic structural variations were evidenced from substantial rise in storage moduli with time in a nonlinear fashion. For instance at 0.1 Hz, the magnitude of *G*′ was initially at 17 kPa (1 h), but increased to 26 kPa (48 h) and finally reached 45 kPa (72 h); likewise at higher frequencies (e.g. 100 Hz) we found *G*′ exceeds a factor of three times higher than that of a freshly prepared sample. Significantly, the loss tangent plot in Figure 3f shows that, independent of frequency, there is no phase change detected beyond 48 h. This finding further substantiates the claim that, over time, weak supramolecular network in **G⊃ACN** gel has become more rigid (higher *G*, see Figure S25 in the Supporting Information) and thus mechanically strengthened against further shear-induced deformation, as it evolves into **VE⊃ACN**, constituting a vastly interwoven microstructure (Figure 2b). Dynamic strain sweep measurement on **VE⊃ACN** has established a strain tolerance of ≈11%, which was determined from the crossover point between the storage and loss moduli (Figure S26, Supporting Information). Moreover, we performed constant-stress creep experiments comparing the strain recovery of samples at 24 h versus 72 h, in which the latter case corresponding to **VE⊃ACN** shows remarkably higher creep resistance and strain recovery response (Figure S27, Supporting Information).

Electrical conductivity measurements were performed under direct current settings using a potential bias of ±10 V. The conductivity data presented in **Figure** **4** show tunable electrical properties, for which the magnitude of charge mobility can be varied for samples synthesized from different solvents. Notably, the sample of **G⊃MEH** exhibits the highest electrical conductivity value at 9.44 S m^−1^, whereas the lowest conductivity was determined for **G⊃DMSO** at 0.14 S m^−1^, i.e., approaching two orders of magnitude below that of the former gel. The second highest conductivity was determined in **G⊃ACN** (2.51 S m^−1^), which is followed by **G⊃ETH** (1.14 S m^−1^). While a relatively low conductivity has been ascertained for **G⊃ACN** (2.51 S m^−1^), surprisingly, the viscoelastic material (**VE⊃ACN**) derived from the same gel sample yields a markedly higher conductivity value of 9.86 S m^−1^ (Figure S29, Supporting Information). Indeed, the unique ability for tuning the electrical properties of MOF materials is currently of great significant interest.[31] Additionally given that **VE⊃ACN** is malleable, displaying good strain tolerance (see Figure S26 in the Supporting Information) and it can be easily shaped or molded into stable structures (Figure 1b), its improved electrical conduction property may potentially find utility in future device integration.[32]

**Figure 4:**
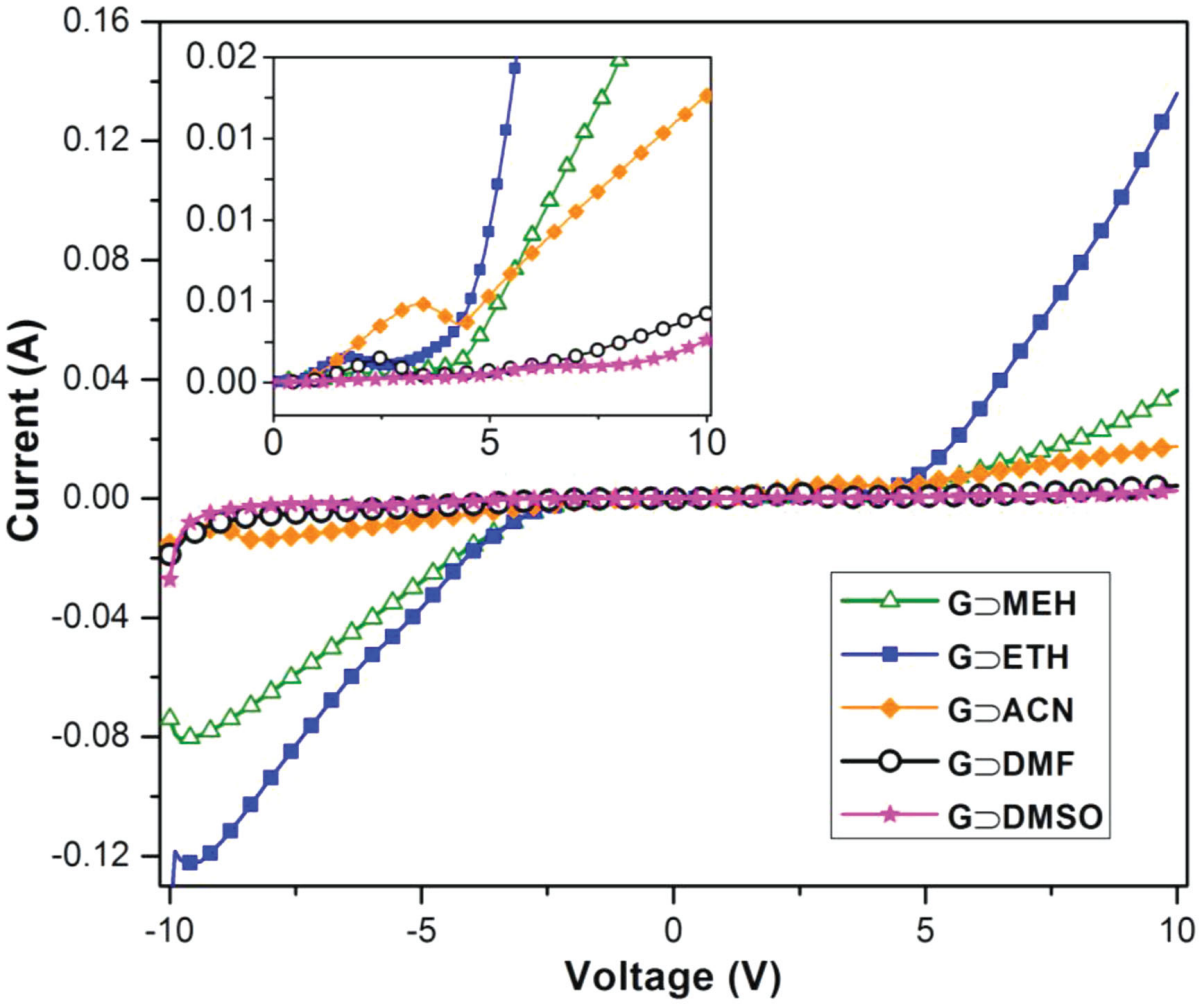
Current versus voltage (*I*–*V*) curves demonstrating tunable electrical conductivities of supramolecular MOF-based hybrid materials synthesized by using different solvents. The inset shows the relatively flat sections between approximately ±5 V, which might be associated with the work function of the aluminum electrodes used in direct current conductivity measurements.

Hydrogen bonding and π–π interactions dominating the fiber network architecture could play an important role in integration of multiple charge carrier species (Figure S29, Supporting Information), which are responsible for facile charge (ionic) transport in the current series of supramolecular MOF hybrids. In situ formation of nano-HKUST-1 particles in the supramolecular assembly also functions as molecular anchors to adjacent fibrous network, in which MOF channels occupied with (guest) charge carrier species will facilitate additional charge transfer. Nevertheless, poorer electrical conductors in the case of, for example, **G⊃DMF** (0.23 S m^−1^) and **G⊃DMSO** (0.14 S m^−1^) could be explained by the “cage effect”[33] of charge carriers (weakly interacting ions), whose mobility is impeded by strongly interacting solvent molecules surrounding the stable gel phase, thus suppressing its overall charge mobility.

In fact, recently Allendorf et al.[34] revealed that electrical conduction mechanism in HKUST-1 can be explained by the extrinsic guest doping mechanism, where tetracyanoquinodimethane (TCNQ) guest molecules interact with Cu(II) sites across the framework voids, forming an uninterrupted linear chain of Cu(paddle wheel)—TCNQ clusters enabling charge transport. Following this line of enquiry, here we propose that electrical conductivity witnessed in the current family of supramolecular fiber networks may be attributed to the integrated effects of weakly interacting charge carrying species, encompassing Cu(II), BTC^3−^, NEt_3_^+^, NO_3_^−^, and HKUST-1 nanoparticles (via active metal sites) bearing charge carriers, all of which are abundant in the hybrid supramolecular assemblies (Figure S29, Supporting Information).

In addition to the unique physical properties of supramolecular MOF hybrids discussed above, another advantage of such sol–gel phases lies in the making of HKUST-1 NMOF thin films. By exploiting hybrid gels as precursors, we have established the applicability of three different techniques to fabricate HKUST-1 films, namely dip coating, doctor blade coating, and spin coating. High uniformity in crystallite size contributes to the generation of compact and high-quality thin films, particularly without having to resort to the use of relatively costly self-assembled monolayer (SAM). For the exemplar in **Figure** **5**, addition of solvent (here methanol) to **G⊃MEH** breaks down rapidly the gel fibrous network to yield pure HKUST-1 nanoparticles (NPs), whose sol suspension can be facilely dip coated onto a glass substrate, silicon wafer etc. Such NP suspension facilitates large-area thin film deposition operation, for instance, sol obtained from **G⊃DMSO** is especially effective for use with the doctor blade technique to produce films with well controlled thicknesses (Figures S30, Supporting Information), which have been characterized via infinite focus microscopy (Figure S31, Supporting Information).

**Figure 5:**
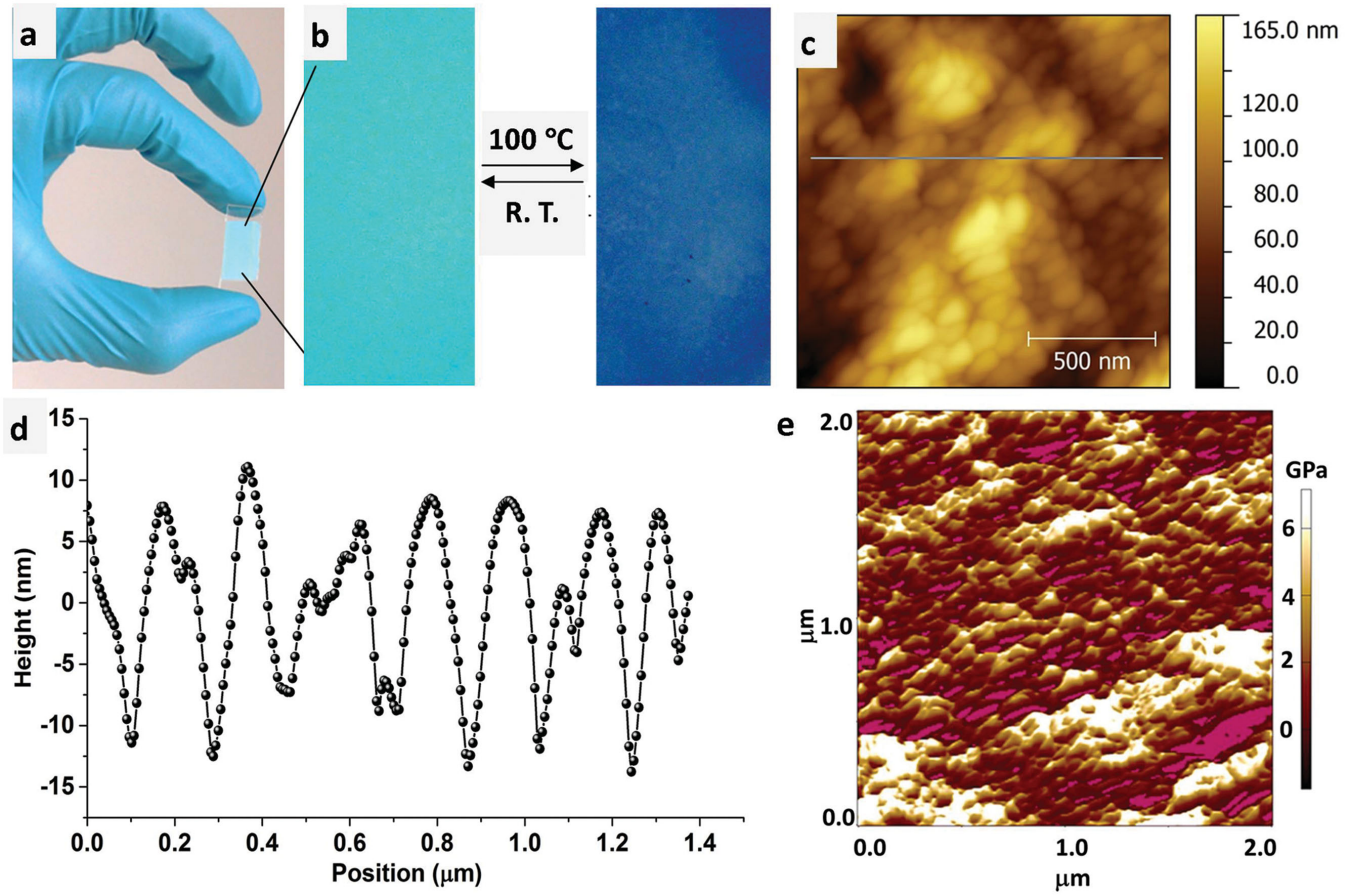
Thin films of HKUST-1 fabricated by dip coating using nanoparticle suspension (sol) of G⊃MEH: a) on a glass substrate, b) thin film demonstrating reversible color change upon heating, c) AFM height topography, d) surface roughness profile extracted from AFM line scan marked in (c). e) Young's modulus stiffness map of the thin film surface measured by AM–FM technique. The wide range of elastic moduli obtained (3–6 GPa) may be linked to elastic anisotropy,[36] suggesting that the external facets of HKUST-1 nanocrystals are oriented across a range of crystallographic directions.

Coated films were air dried and then washed by careful immersion into methanol for ≈15 min; this step removes unwanted soluble parts thus leaving behind pure HKUST-1 NMOF films (Figures S12, Supporting Information). Unwashed coatings show green color upon heating to higher temperature, while the washed version shows color change expected of an activated HKUST-1 material, i.e., switching from turquoise to dark blue (Figure 5b) upon removal of coordinated water from the copper centers within the porous framework. Thin films produced by sol–gel approach display excellent uniformity with typical surface roughness of ≈10–30 nm and a mean grain size of ≈100 nm, as determined by AFM surface topo­graphy (Figure 5c,d). Furthermore, the high sensitivity of AFM is ideal for characterizing fine-scale mechanical properties of thin film surfaces. In a first experiment, we attempted the combined AM–FM approach (amplitude modulated–frequency modulated)[35] using high-resolution amplitude tapping mode (topo­graphy by AM) and high-sensitivity frequency modulation mode (elasticity by FM) to interrogate the nanomechanical behavior of HKUST-1 coatings. Figure 5e shows the elastic stiffness map of HKUST-1 thin film, where the Young's modulus (*E*) was found to lie between ≈3–6 GPa; this stiffness magnitude is broadly consistent with elastic constants measured for a wide range of porous MOF materials, where *E* < 10 GPa.[36] Herein, we demonstrate that the AM–FM approach recently implemented in an AFM setup is promising for studying nano­mechanical properties of MOF thin films, which otherwise will be inaccessible to other mechanical characterization techniques.

Novel development of multifunctional materials is ultimately dictated by precise control over its fine-scale microstructural organization, made possible through the mechanism of molecular self-assembly. Indeed, it is central to establish the collective effects underpinning complex structural evolution, specifically to gain insights into structure–property relationships across the multiple temporal and length scales. In this work, we illustrate that an unconventional system comprising MOF-based supramolecular hybrid gels can be created via hierarchically self-organizing inorganic and organic-basic building blocks. The highly tunable microstructural features illustrated in this study are distinctively different from the “traditional” hybrid gels and soft matter. By leveraging the synergistic formation of highly aligned fiber networks coexisting with porous MOF nanoparticles, this work offers an exciting new strategy to tailor multistimuli-responsive properties, yielding coupled chemical, electrical, structural, and mechanical characteristics, all of which are key to sensors, actuators, and many other thin-film microelectronics and advanced device technologies. The novel viscoelastic material (**VE⊃ACN**) derived from a hybrid gel is also worth highlighting, given its excellent combination of strain damage tolerance, dynamic mechanical recovery, and enhanced electrical conductivity. Finally, by virtue of the immense chemical and structural configurations of MOFs,[11a-c] it is conceivable that the facile synthetic methodology we illustrated here provides the basis for rapid adaptation to many wide-ranging hybrid systems, opening up exciting possibilities of connecting the emerging field of MOFs with the vibrant disciplines of supramolecular gels and soft matter.

## Experimental Section

*Materials Synthesis*: 1,3,5-Benzenetricarboxylic (BTC) acid (2 mmol) was dissolved in respective solvents and then triethylamine (6 mmol) was added in order to achieve complete soluble ligand in the solvent. This solution was sonicated for 5 min prior to reaction with the copper nitrate solution, which was prepared in a corresponding solvent by taking 3 mmol of salt and dissolving further with sonication for 1–2 min. Solution of copper nitrate was then added to 1,3,5-benzenetricarboxylic acid with vigorous shaking for a few seconds, the mixture is then left undisturbed till it shows gel-like behavior as confirmed by the tube inversion method.

*Thin Film Fabrication*: Resultant hybrid gel materials obtained from the aforementioned synthesis were used as precursors for fabricating MOF thin films. Each material was washed three times using 20 mL of methanol and then centrifuged to collect the NMOF particles. Suspension remained at the bottom of centrifuge tube was collected and later used to deposit thin films of MOF onto glass substrates via the doctor blade technique, with varying gap size set between the tip of the blade and surface of the glass substrate [from a few micrometers (4 μm), up to 10s of micrometers (50 μm)]. The same NMOF suspension has also been successfully used for dip coating and spin coating methods [step wise: (i) 500 rpm for 50 s, (ii) 800 rpm for 50 s, and (iii) 1000 rpm for 20 s].

*Materials Characterization*: Rheological measurements were performed on the Physica MCR-301 (Anton Paar) rheometer equipped with a temperature-controlled basal plate. Parallel plate configuration was used for all studies by keeping a 1 mm gap distance between the basal and the top plates. A constant shear stress of 10 Pa was applied for creep and stress recovery tests. Gel specimens were coated with a thin layer of gold using the SC7620 Polaron sputter coater (Quorum Technologies) before examination under the scanning electron microscope (Carl Zeiss EVO LS15). Optical images and surface height topography of MOF thin films were characterized using infinite focus microscopy, IFM (Alicona InfiniteFocus 3D profilometer). X-ray powder diffraction characterization of nanoparticles and gel samples were performed using the Rigaku Smart Lab diffractometer with Cu Kα source (1.541 Å); diffraction data were collected at 2*θ* angle from 2° to 30°, using a 0.01° step size and 1° min^−1^ step speed. Atomic force microscopy (AFM) height topography and AM–FM tapping mode imaging were carried out using the Asylum Research MFP-3D AFM in air. Silicon AFM probe (Tap300-G, Budget Sensor) with resonant frequency of 300 kHz and a force constant of 40 N m^−1^ mounted on the AM–FM cantilever holder was used for nanomechanical characterization; the tip calibration was performed using a standard sample of Matrimid5218 with an established Young's modulus (*E* = 4 GPa). Electrical conductivity of gel samples was measured using the Keithley 2614B sourcemeter and a custom-designed conductivity cell lined with aluminum electrodes spaced at 1 cm apart. The conductivity of the viscoelastic solid was measured from a thin layer of **VE⊃ACN** material sandwiched between a pair of flat aluminium electrodes.

## Supporting Information

As a service to our authors and readers, this journal provides supporting information supplied by the authors. Such materials are peer reviewed and may be re-organized for online delivery, but are not copy-edited or typeset. Technical support issues arising from supporting information (other than missing files) should be addressed to the authors.
