# Supplementary material for: Multifunctional Supramolecular Hybrid Materials Constructed from Hierarchical Self-Ordering of In Situ Generated Metal-Organic Framework (MOF) Nanoparticles
Source: Adv Mater. 2015 Jun 25;27(30):4438–46. doi: 10.1002/adma.201501448 (PMC4584506; doi:10.1002/adma.201501448)
Supplement: Supplementary file 1 — Supplementary [file adma0027-4438-sd1.pdf]

Copyright WILEY-VCH Verlag GmbH & Co. KGaA, 69469 Weinheim, Germany, 2015.

ADVANCED MATERIALS

Supporting Information

for *Adv. Mater.,* DOI: 10.1002/adma.201501448

Multifunctional Supramolecular Hybrid Materials Constructed from Hierarchical Self-Ordering of In Situ Generated Metal-Organic Framework (MOF) Nanoparticles

*Abhijeet K. Chaudhari, Intaek Han, and Jin-Chong Tan**

Copyright WILEY-VCH Verlag GmbH & Co. KGaA, 69469 Weinheim, Germany, 2013.

Supporting Information

Multifunctional supramolecular hybrid materials constructed from hierarchical self-ordering of *in situ* generated metal-organic framework (MOF) nanoparticles

Abhijeet K. Chaudhari,[1] *Intaek Han*[2] *and Jin-Chong Tan*[*,1]

[1]*Department of Engineering Science, University of Oxford, Parks Road, Oxford OX1 3PJ, United Kingdom*
[2]*Materials Research Center, Samsung Advanced Institute of Technology (SAIT), Samsung Electronics, Suwon 443-803, Republic of Korea*

[*]*E-mail*: jin-chong.tan@eng.ox.ac.uk

Table of Contents

Scheme: Supramolecular MOF hybrids 2
Table S1: Base-dependent gelation in different solvents 4
Table S2: Sol-gel transformation *via* chemical stimuli in G⊃DMSO system 5
Scanning electron microscopy (SEM) images 6
Hydrogen bonding and π-π interactions 12
Powder X-ray Diffraction (PXRD) 15
Atomic force microscopy (AFM) of HKUST-1 nanoparticles 16
Investigating the fibre formation mechanisms 17
Rheological studies 20
Electrical conductivity measurements 27
MOF thin films fabricated from sol-gel method 28
References 30

Scheme: Supramolecular MOF hybrids

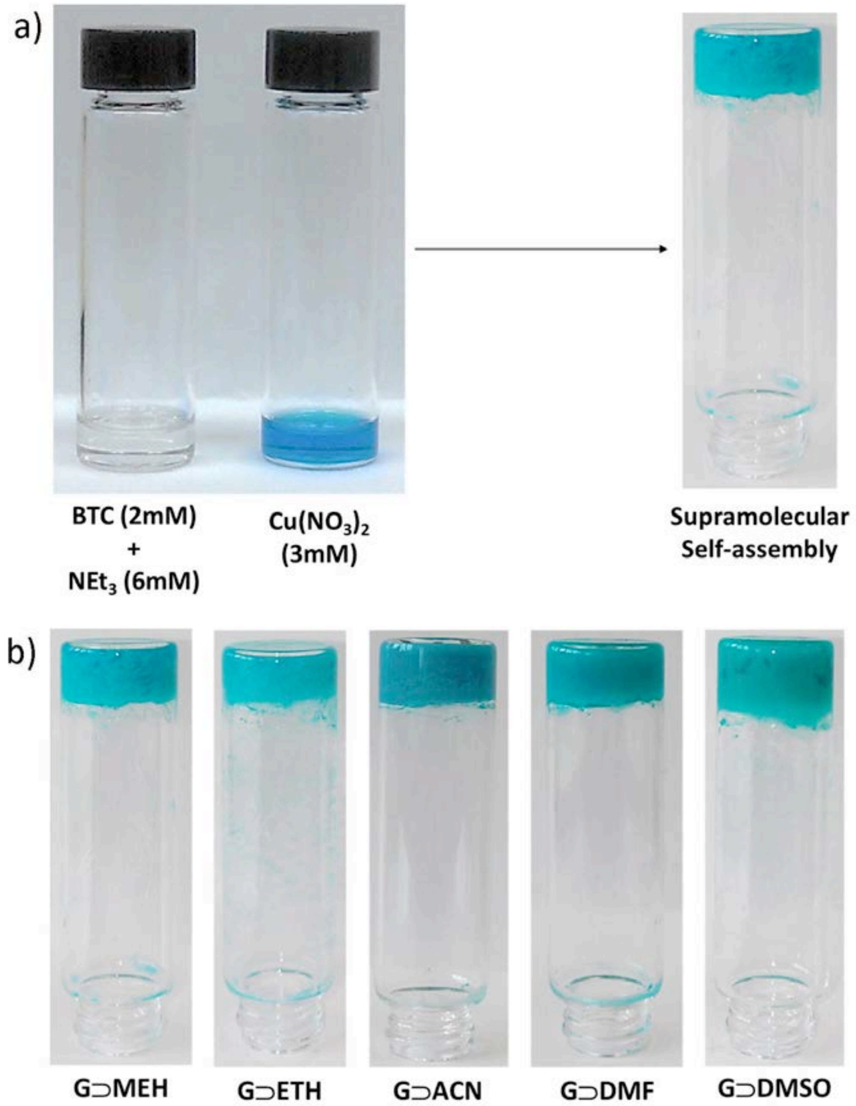

Figure S1. a) Scheme showing synthesis of the MOF-based supramolecular hybrid self-assembly, b) gel (G) samples synthesized by using different solvents (MEH: methanol, ETH: ethanol, ACN: acetonitrile, DMF: N,N-dimethylformamide, DMSO: dimethyl sulfoxide)

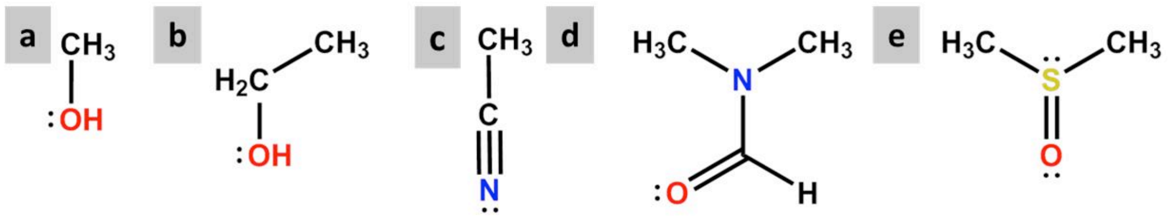

Figure S2. Solvent molecules used for fabricating supramolecular MOF hybrids:
(a) methanol (b) ethanol (c) acetonitrile (d) N, N-Dimethylformamide (e) dimethyl sulfoxide.

Table S1: Base-dependent gelation in different solvents

Solvent ⬇	Base ➡	†NEt_3	NaOH	KOH
Methanol (MEH)		Gel (2 min)	Precipitate	Precipitate
Ethanol (ETH)		Gel (5 min)	Precipitate	Precipitate
Acetonitrile (ACN)		Gel (5 min)	Precipitate	Precipitate
N,N-Dimethylformamide (DMF)		Gel (10 min)	Precipitate	Precipitate
Dimethylsulfoxide (DMSO)		Sol-Gel (20 min)	Precipitate	Precipitate

†Gelation time quoted in brackets

Table above shows that MOF gelation was unsuccessful when using NaOH or KOH as bases, in which precipitation product was obtained instead of a gel.

Table S2: Sol-gel transformation *via* chemical stimuli in G⊃DMSO system

Sol-Gel Cycle #	Addition	Concentration	Time taken for gelation
1	$Cu(NO_3)_2$	0.5 mM	(Sol)
	BTC^{3-}	1.5 mM	(Gel) ~15 min
2	BTC^{3-}	1.5 mM	(Sol)
	$Cu(NO_3)_2$	0.5 mM	(Gel) ~60 min
3	$Cu(NO_3)_2$	0.5 mM	(Sol)
	BTC^{3-}	1.5 mM	(Gel) ~150 min

Table above shows 3 repeated cycles demonstrating sol-gel chemico-response triggered by sequential of Cu(II) or BTC^{3-}. It was found that each subsequent cycle takes approximately twice as long the time needed, compared to the former cycle, to reach complete gelation.

Scanning electron microscopy (SEM) images

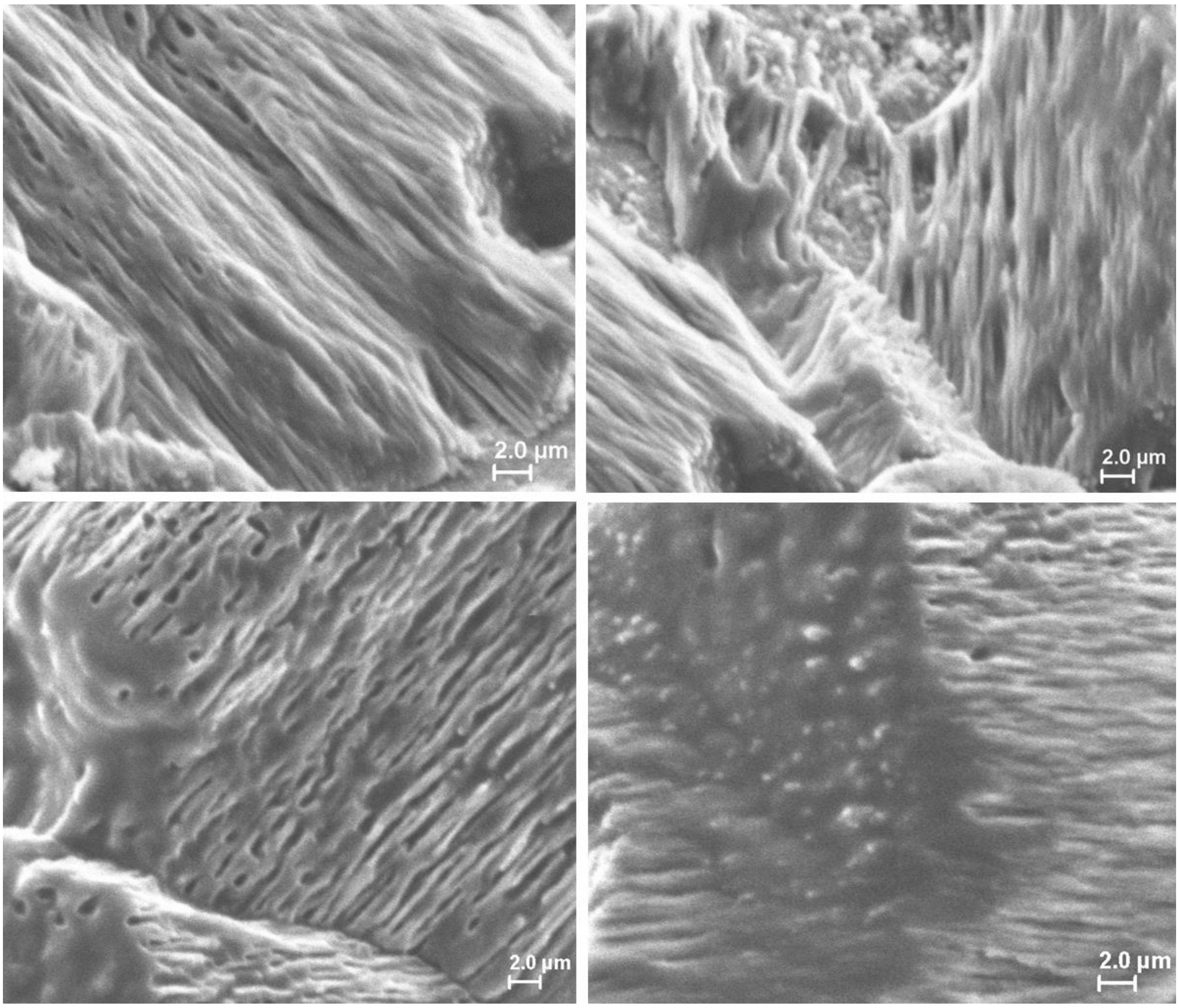

Figure S3. SEM images of visco-elastic solid (**VE⊃ACN**) obtained from **G⊃ACN** assembly, revealing microstructures comprising lamellar fibrous filaments incorporating HKUST-1 nanoparticles.

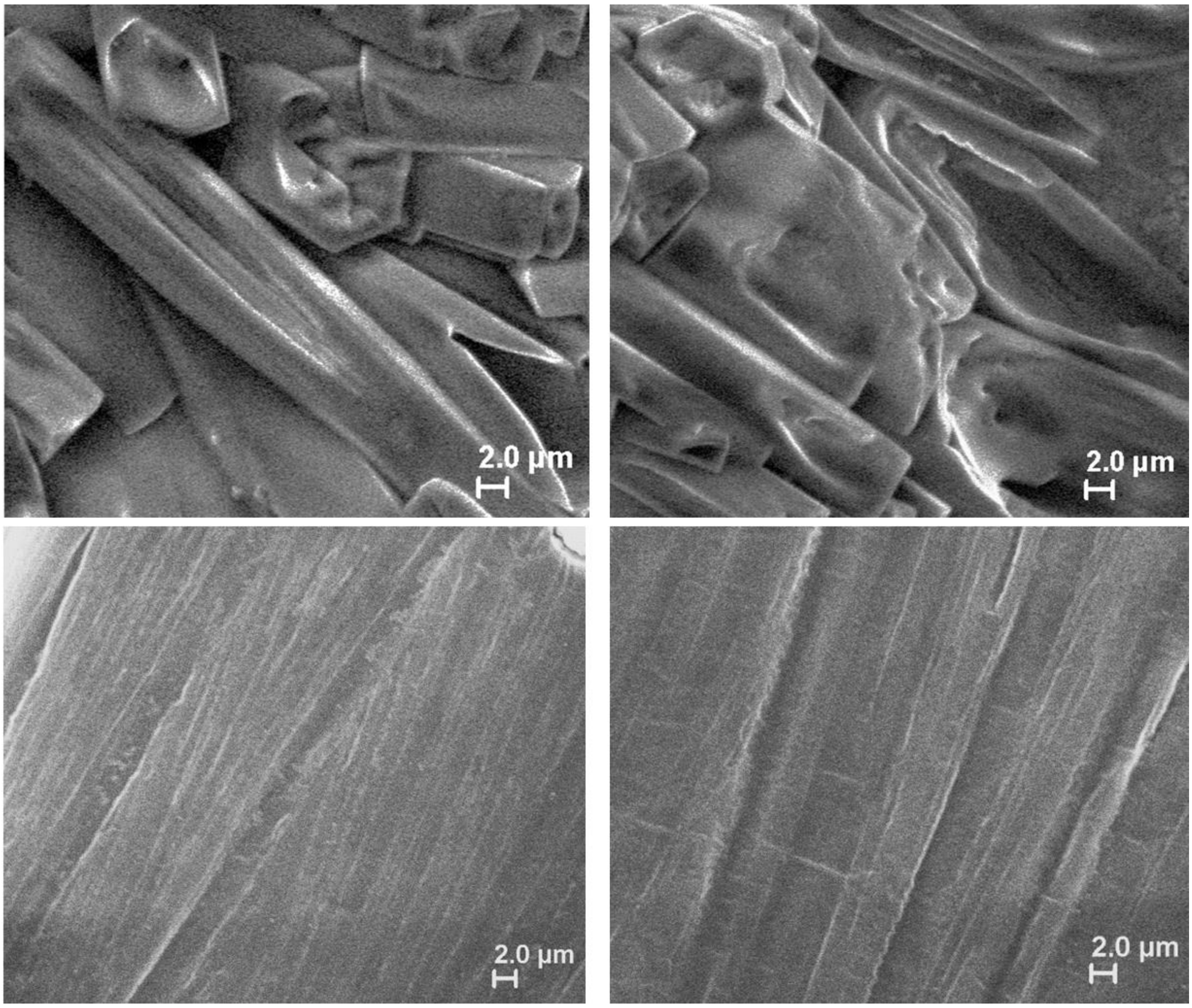

Figure S4. SEM images of dried sample of **G⊃MEH** with lamellar nano-crystalline particle microstructure within the fibrous network.

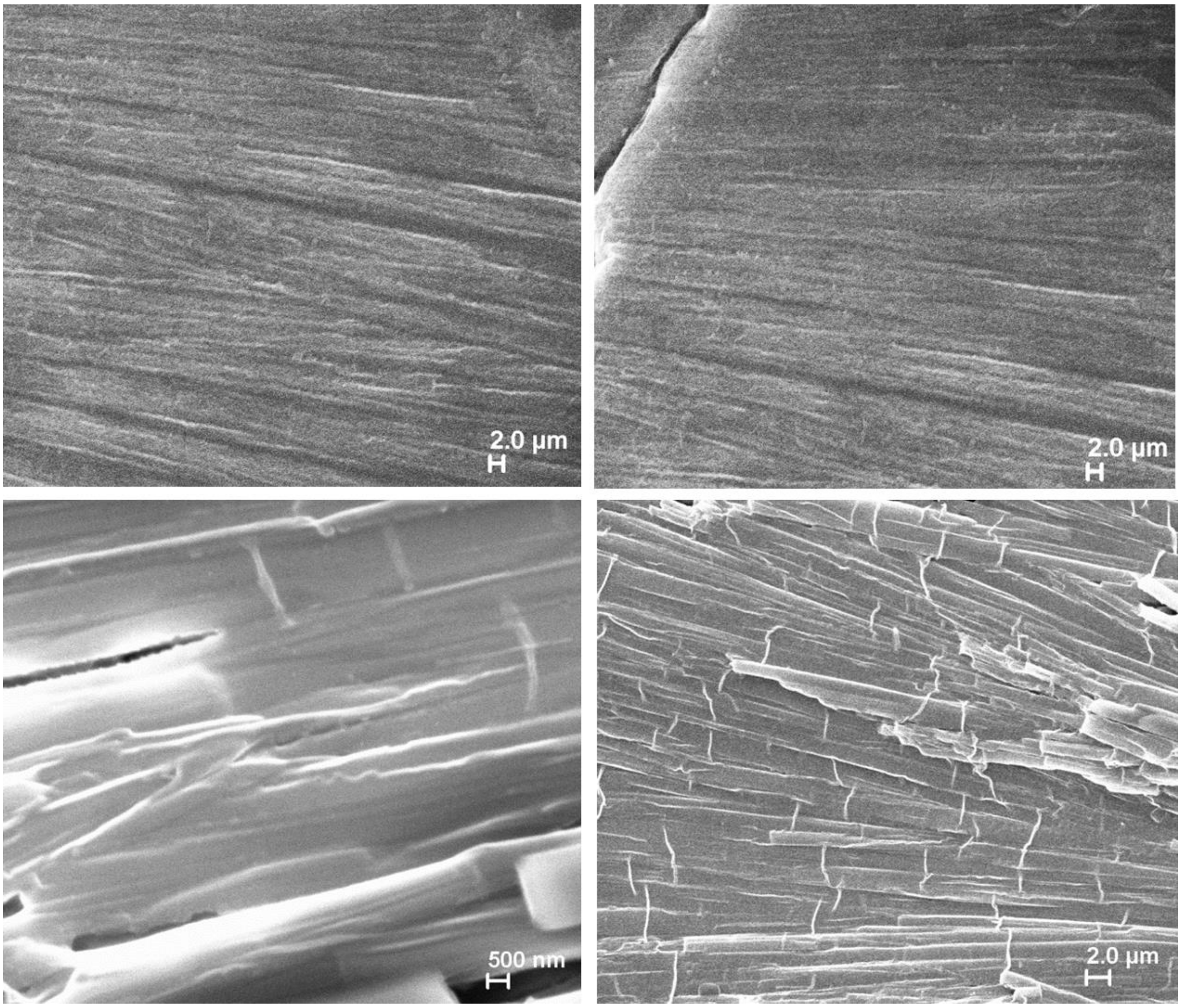

Figure S5. Additional SEM images of dried samples of **G⊃MEH** showing developments of transverse cracking across fibres upon drying of the gel hybrid material.

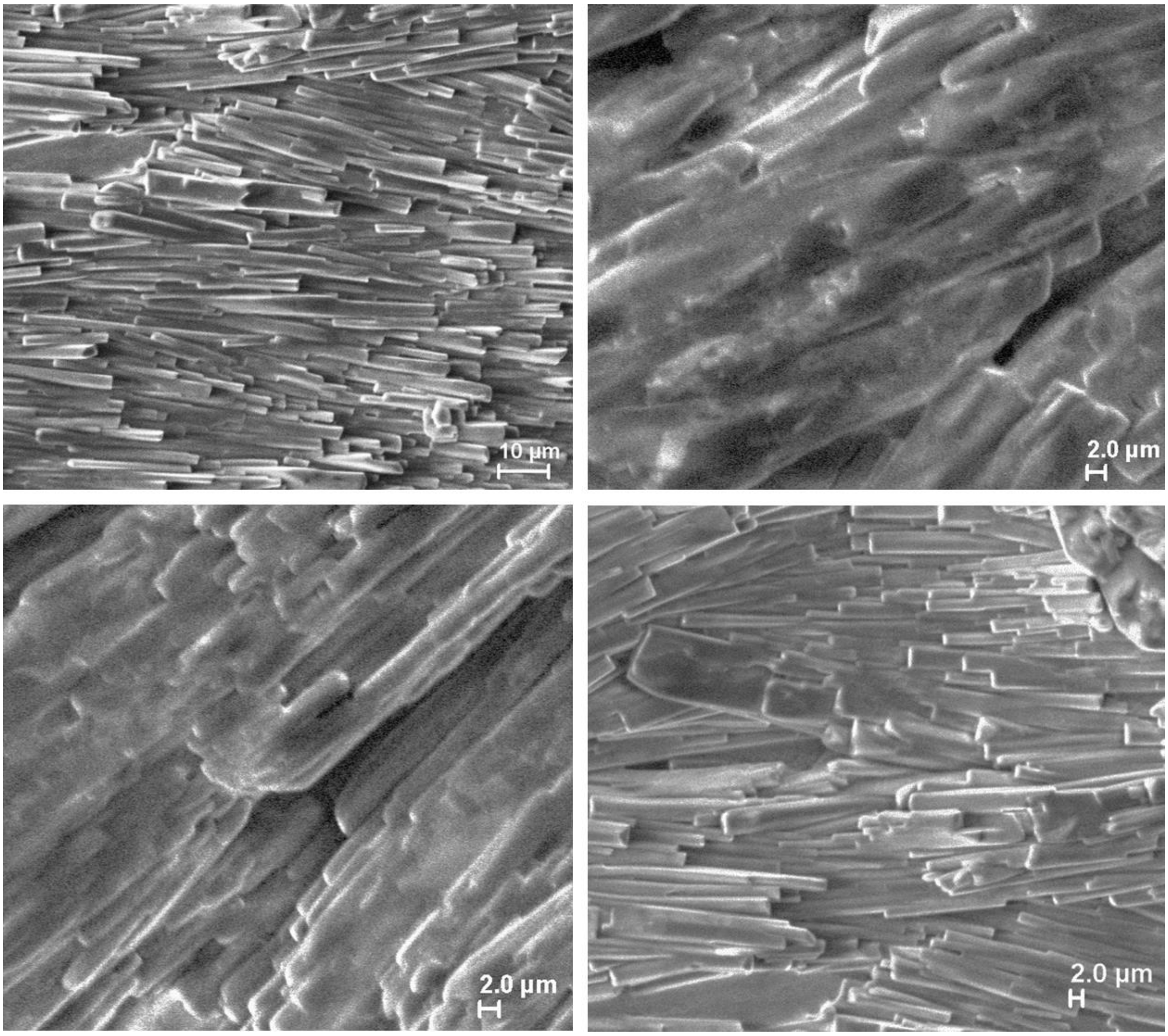

Figure S6. SEM images of dried sample of **G⊃DMF**. Top right and bottom left images show nano-crystalline particle microstructure visible in the fibrous network.

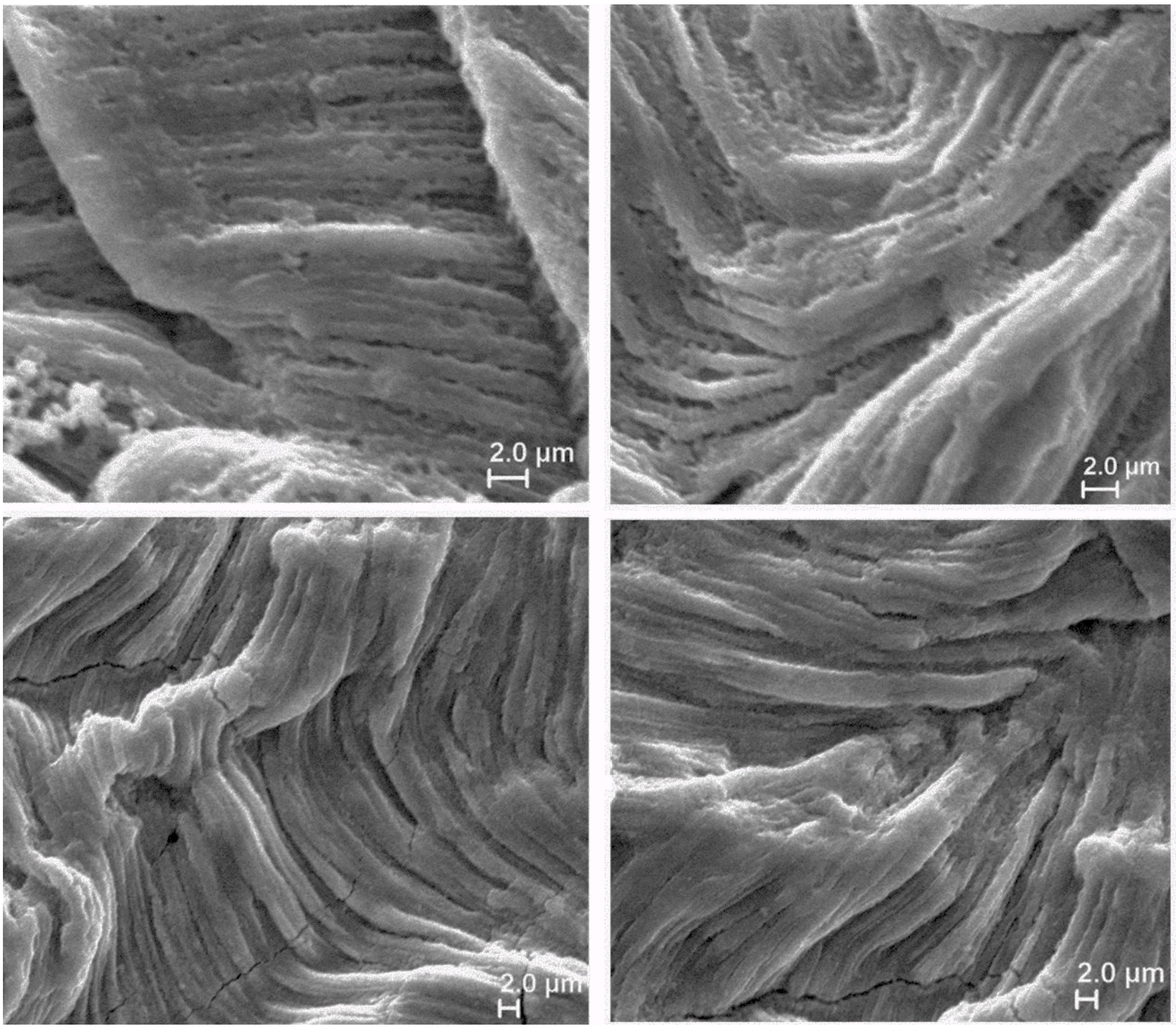

Figure S7. SEM images of dried sample of **G⊃ETH** showing bundles of micron-size fibers, each of which containing HKUST-1 nanoparticles. Note that the transverse cracks developed upon drying of the gel hybrid.

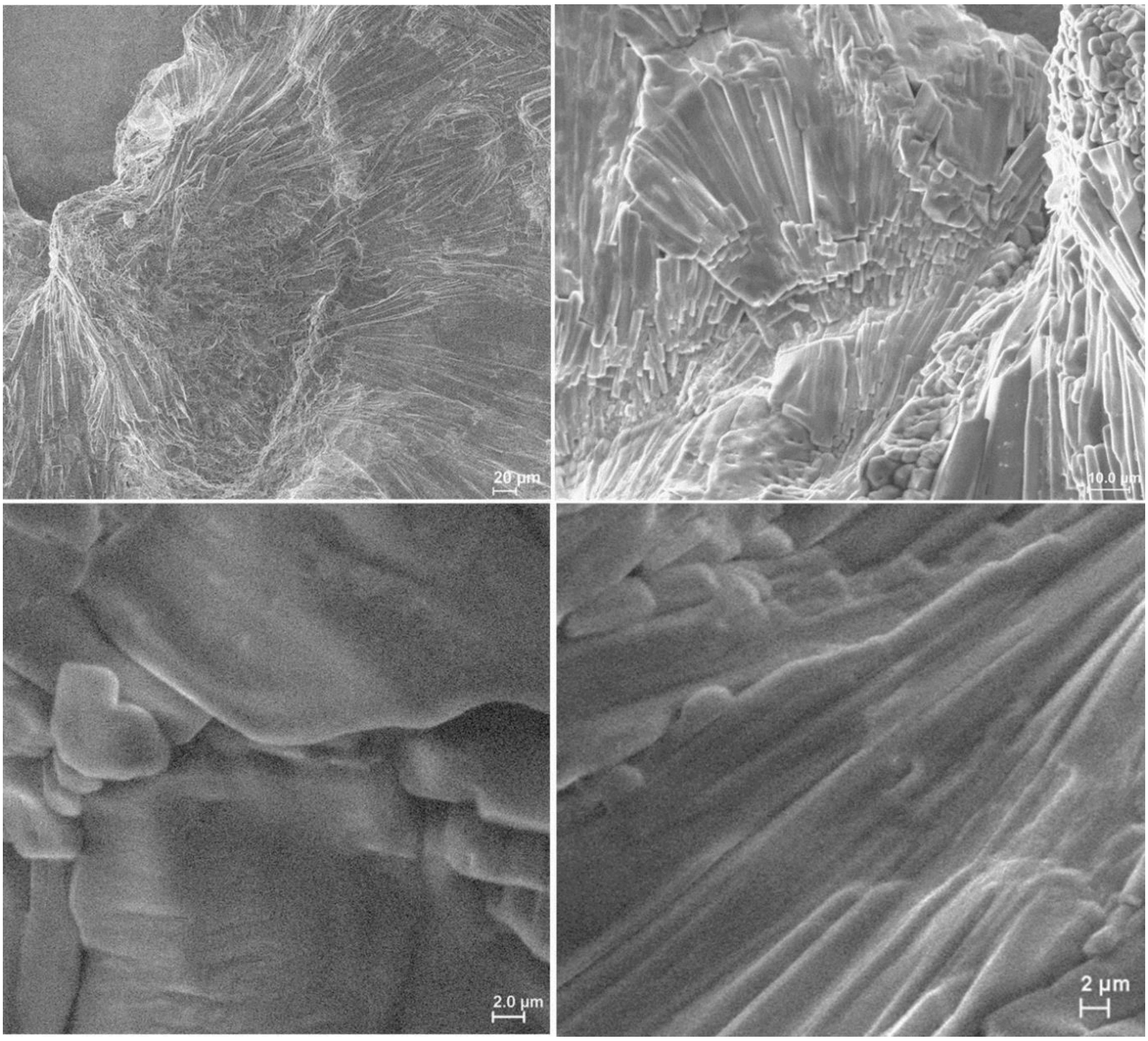

Figure S8. SEM images of dried sample of bulk **G⊃ETH** revealing bundles of fiber morphology formed by self-assembly.

Hydrogen bonding and π-π interactions

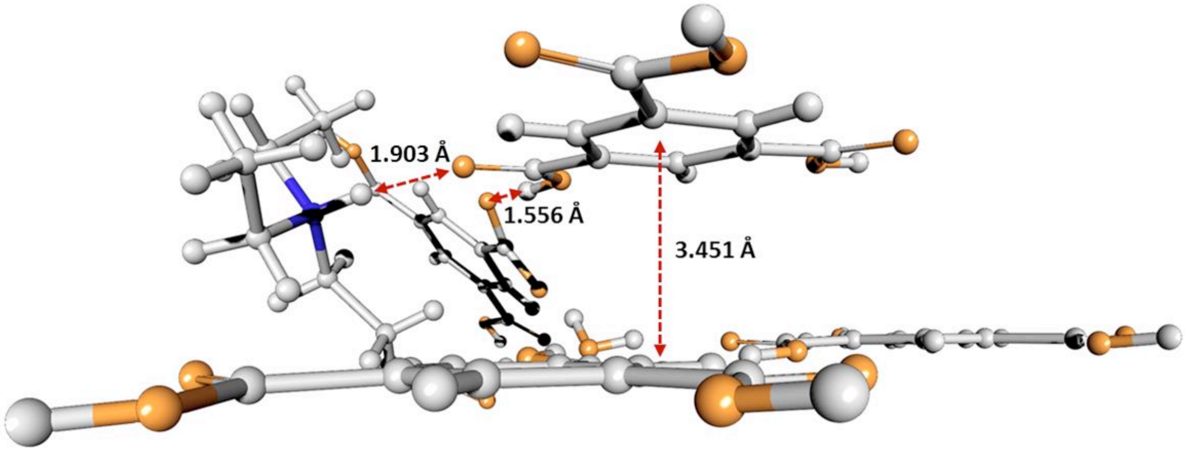

Figure S9. Strong π-π interactions and hydrogen bonding interactions present between the BTC^{3-} and NEt_3 molecules.[2] Color scheme adopted - Blue: nitrogen; Brown: oxygen, Grey: carbon.

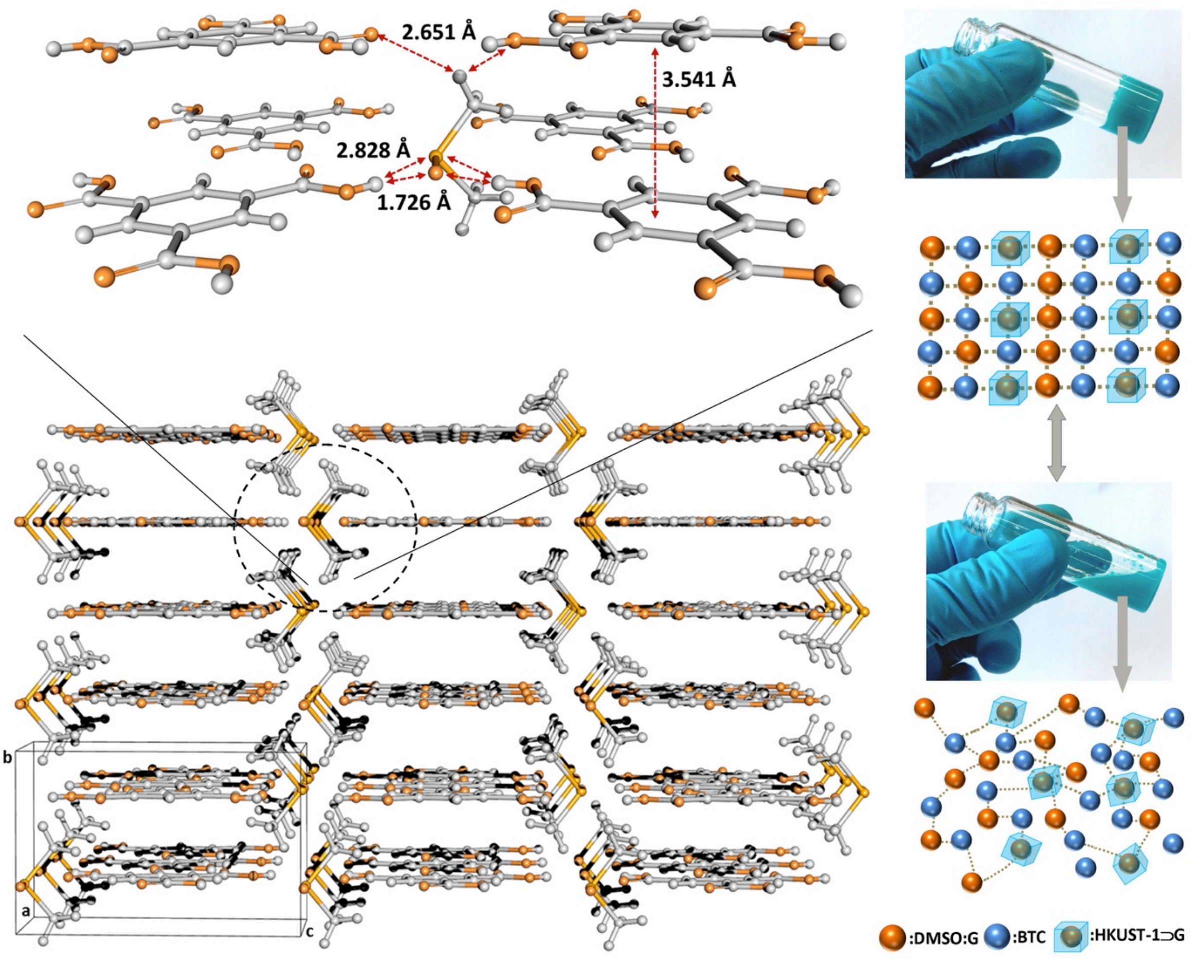

Figure S10. Packing diagram of co-crystals of BTC^{3-} and DMSO molecules showing very strong interactions of S and O atoms with surrounding and aligned layers of BTC^{3-} by means of π-π and hydrogen bonding interactions.[3] Right panel illustrates the proposed reversible sol-gel conversion evidenced in **G⊃DMSO**. Color scheme - Brown: oxygen; Yellow: sulphur; Grey: carbon.

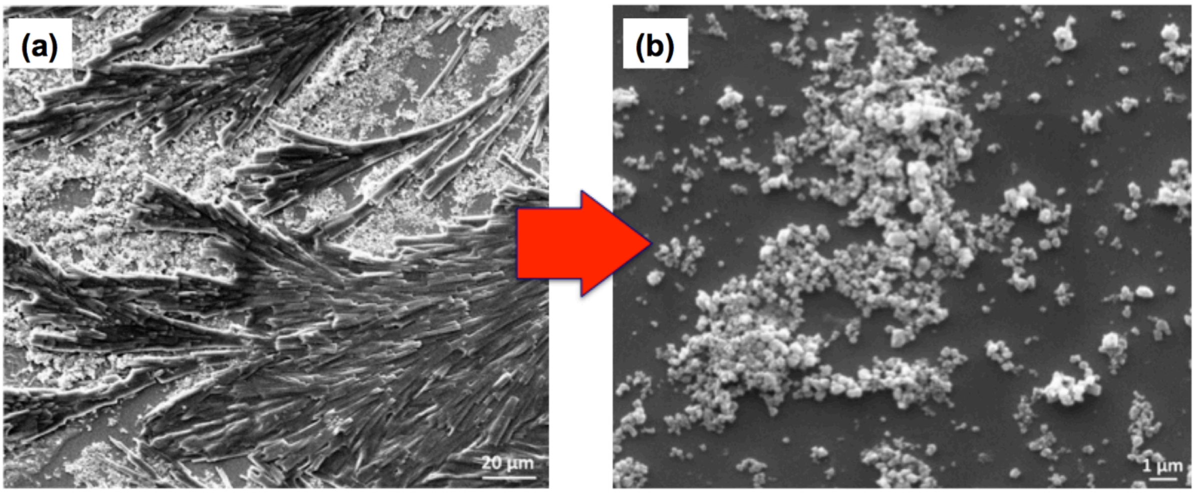

Figure S11. (a) Drying of gel fibrous assembly at room temperature transforms gels into globular nano size particles of HKUST-1. (b) Subsequent immersion into a polar solvent (e.g. MeOH) for less than 1 min causes further breakdown of fibrils to yield pure HKUST-1 nanocrystals (see Suppl. Fig. S13) as final stable product.

Powder X-ray Diffraction (PXRD)

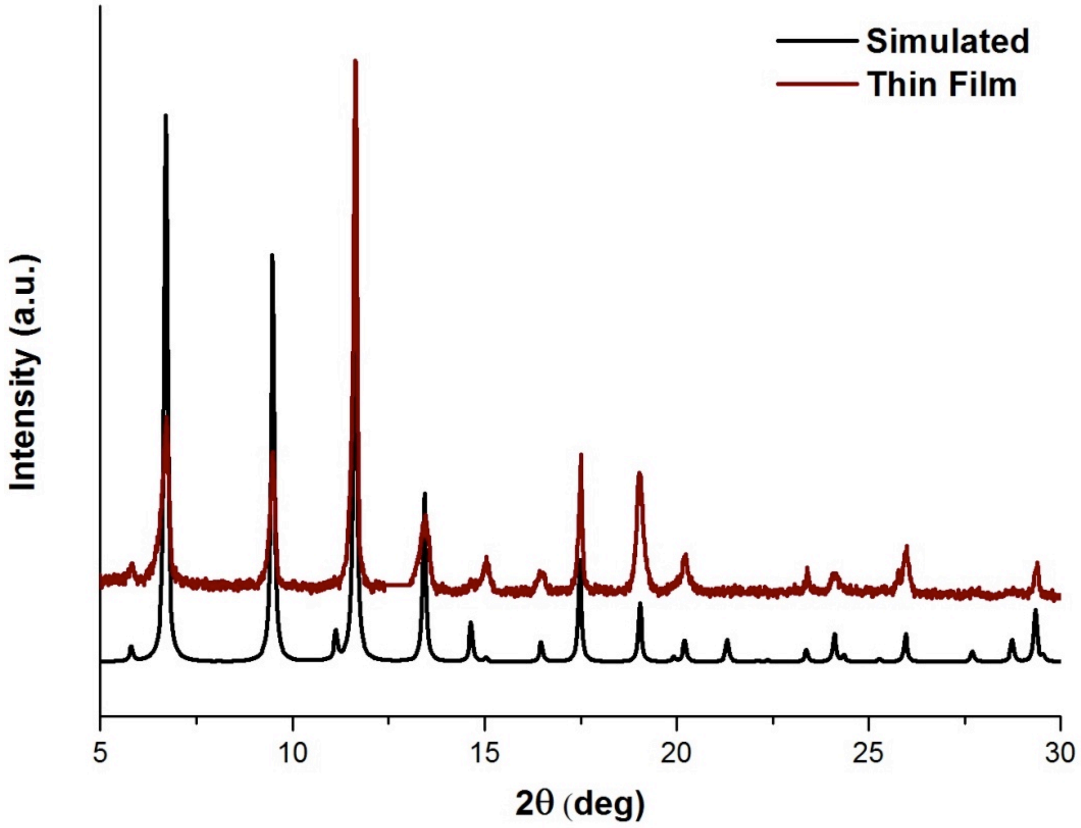

Figure S12. Powder X-ray diffraction patterns for the simulated HKUST-1 (black) and that obtained from the thin film of HKUST-1 (wine) fabricated *via* sol-gel method.

Atomic force microscopy (AFM) of HKUST-1 nanoparticles

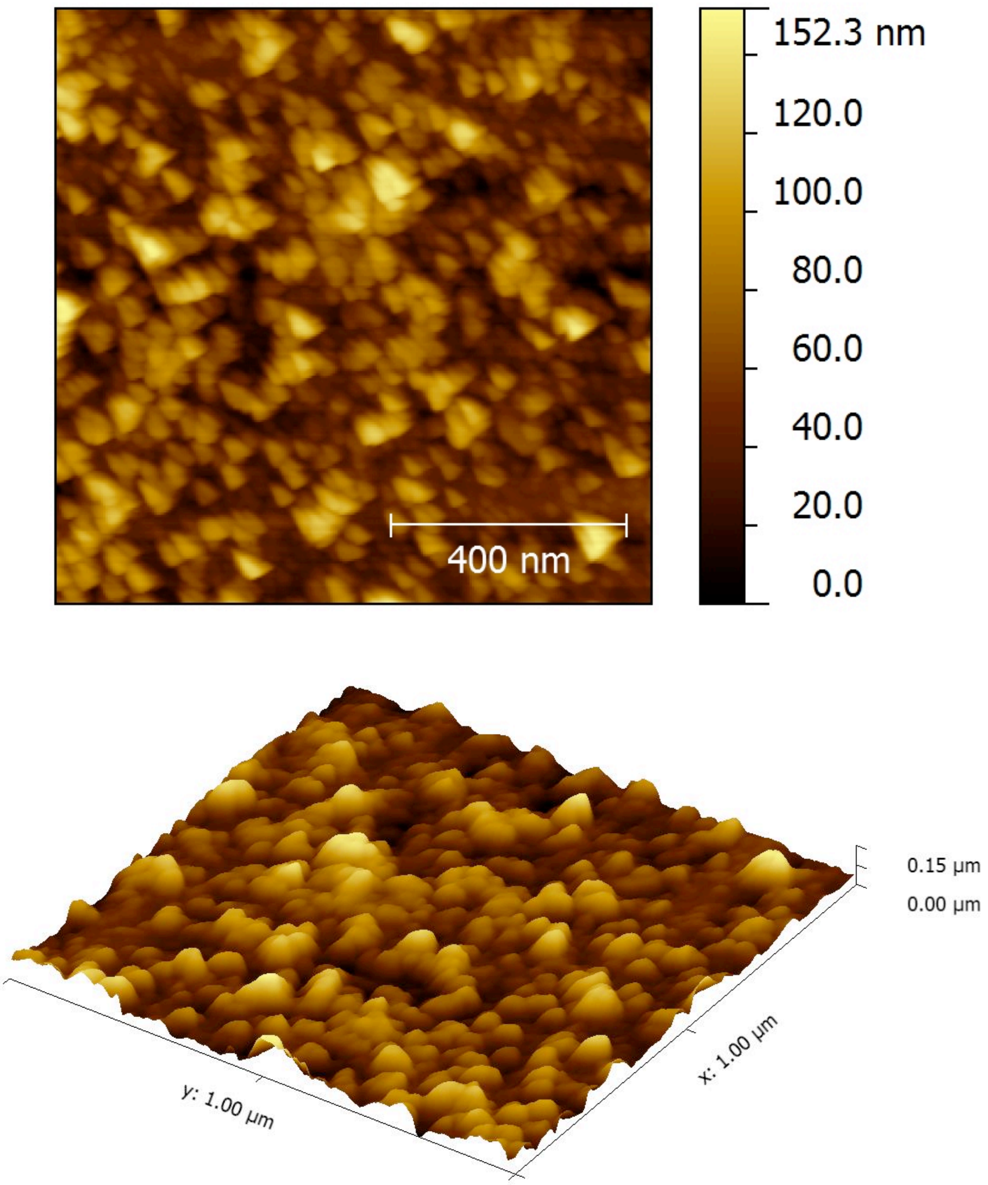

Figure S13. Atomic force microscopy (AFM) imaging of HKUST-1 nanoparticles harvested from the gel-like hybrid materials (top: 2D topography, bottom: 3D view).

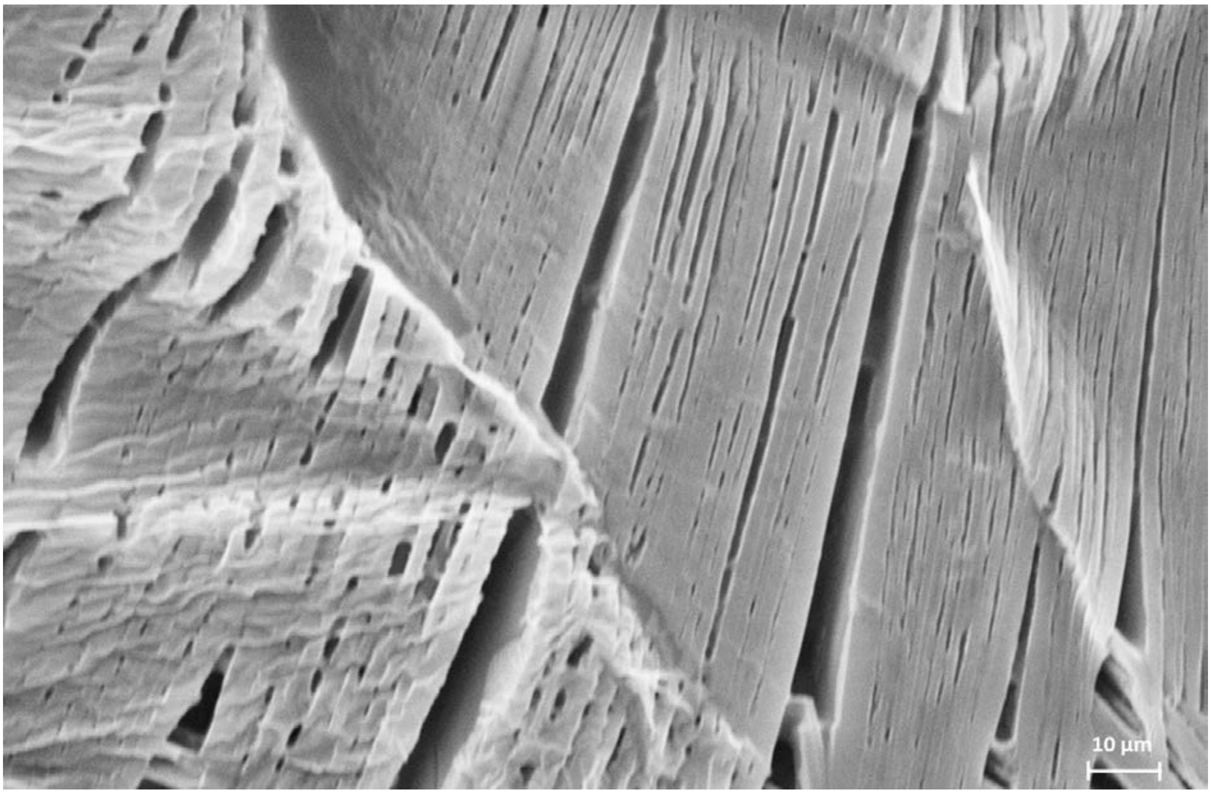

Figure S14. SEM image of a dried sample of **G⊃MEH** obtained by layering two reactants one above the other. The lamellar microstructure is prevalent.

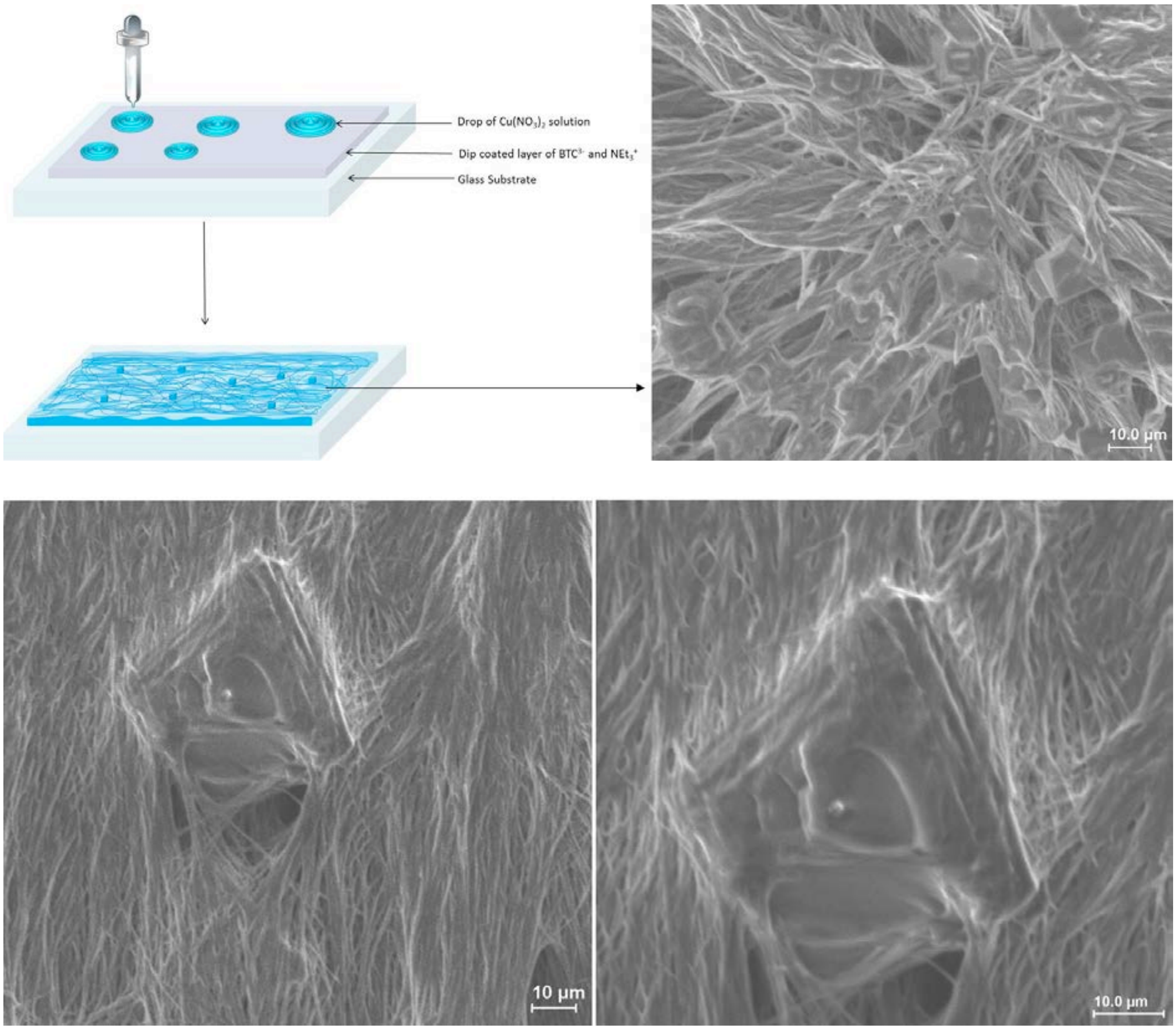

Figure S15. Schematic presentation showing the method adopted for uneven (non-uniform) dissemination of two reactants to understand plausible formation of hybrid gel reaction products. SEM images show that, on top of the fibrous network of **G⊃DMSO** are partially embedded what appears to be a 'defective' HKUST-1 crystal featuring surface steps akin to screw dislocations.[1]

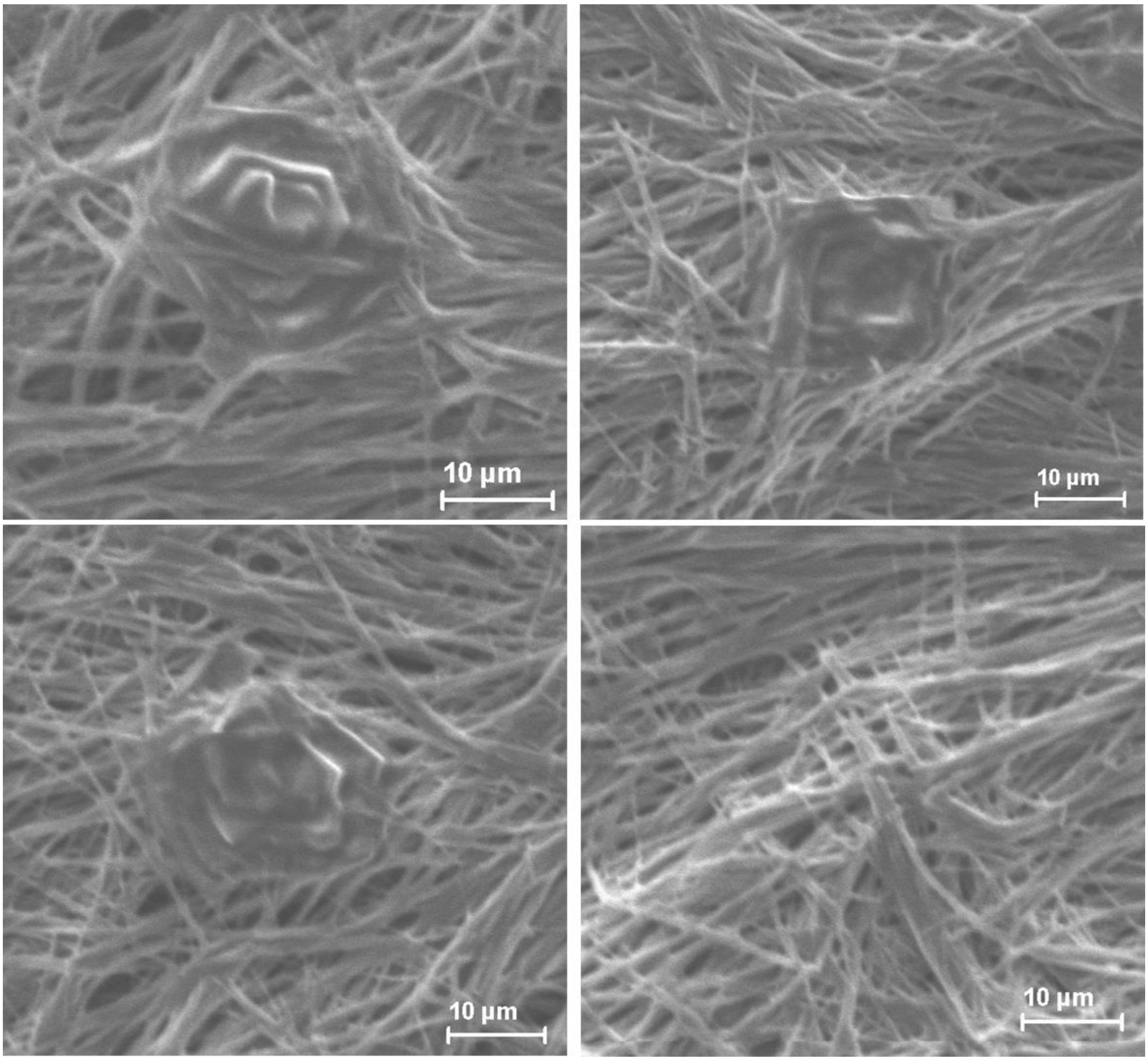

Figure S16. SEM images of the sample made using uneven mixing of reactants showing generation of defective HKUST-1 crystals measuring 10s of microns, from hybrid self-assembly that generates supramolecular MOF hybrid of **G⊃DMSO**.

Rheological studies

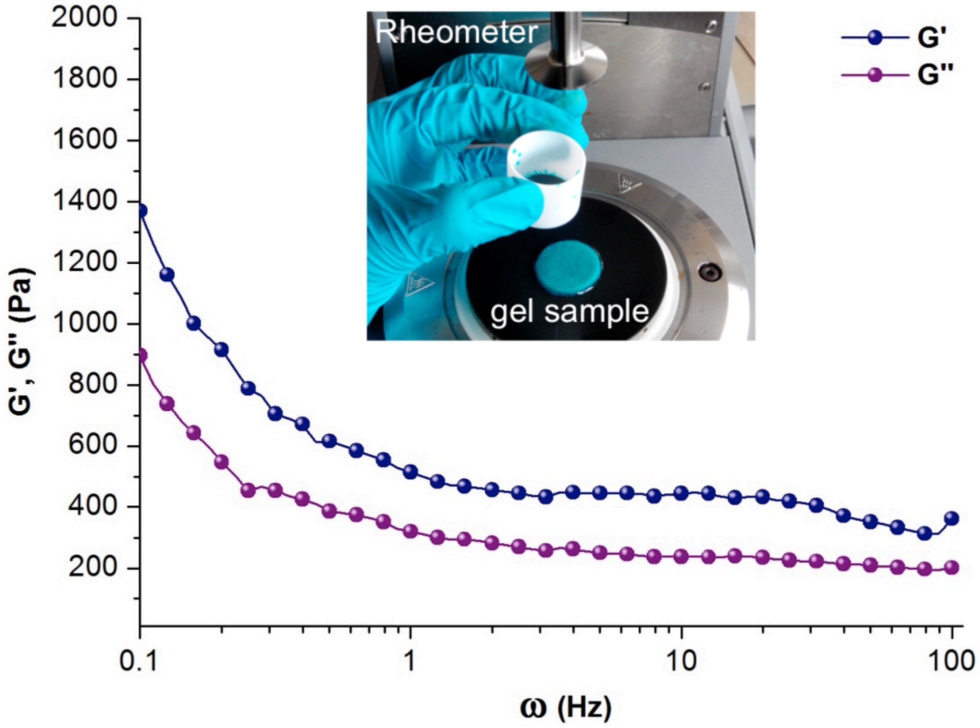

Figure S17. Storage modulus (G′) and loss modulus (G″) of **G⊃MEH** at constant 1% strain. Inset shows the method used for preparing hybrid gels directly on the stage of the rheometer.

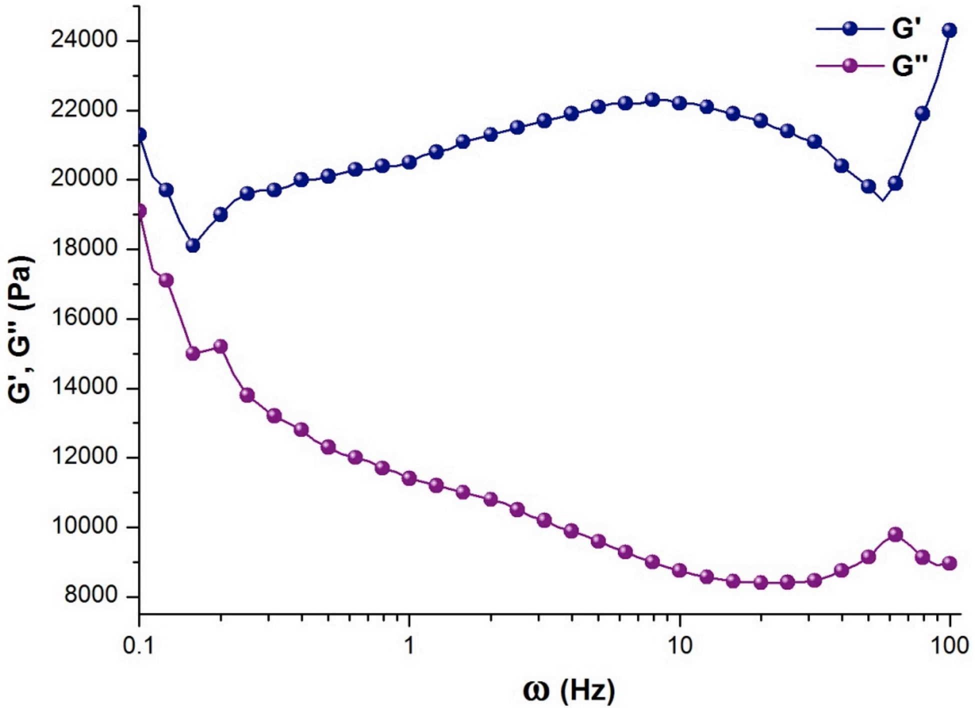

Figure S18. Storage modulus (G′) and loss modulus (G″) of **G⊃ETH**.

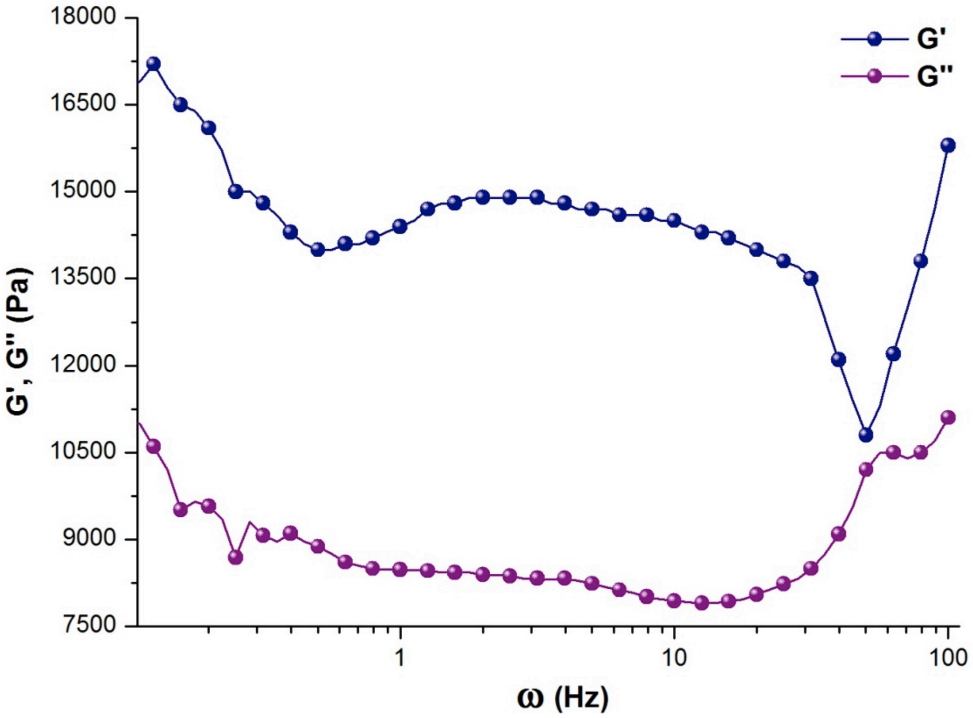

Figure S19. Storage modulus (G′) and loss modulus (G′′) of **G⊃ACN**.

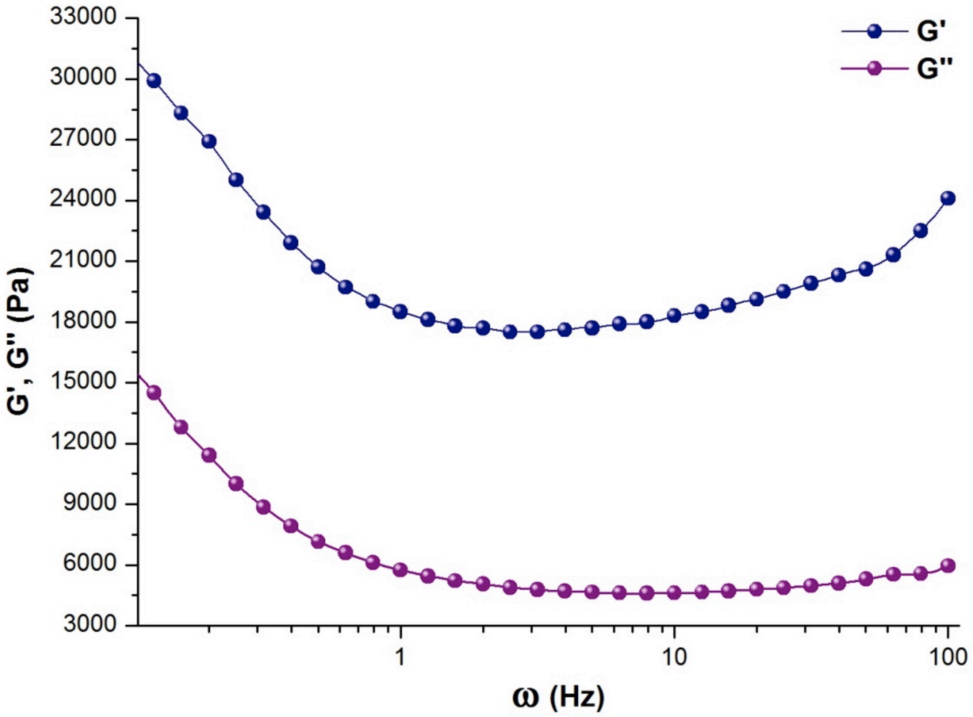

Figure S20. Storage modulus (G′) and loss modulus (G′′) of **G⊃DMF**

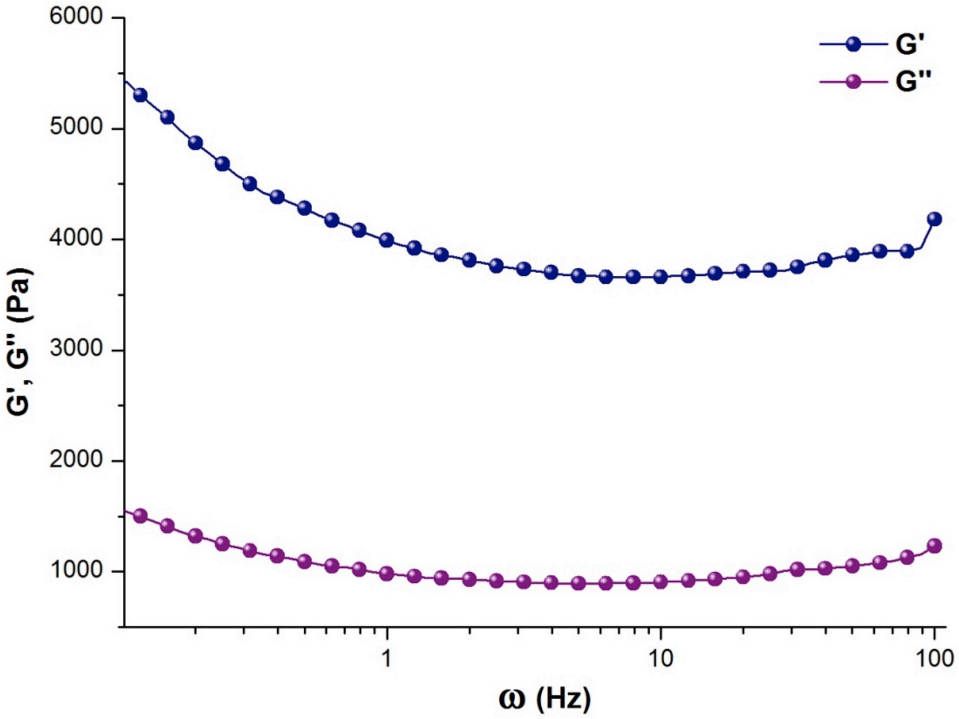

Figure S21. Storage modulus (G′) and loss modulus (G′′) of **G⊃DMSO**.

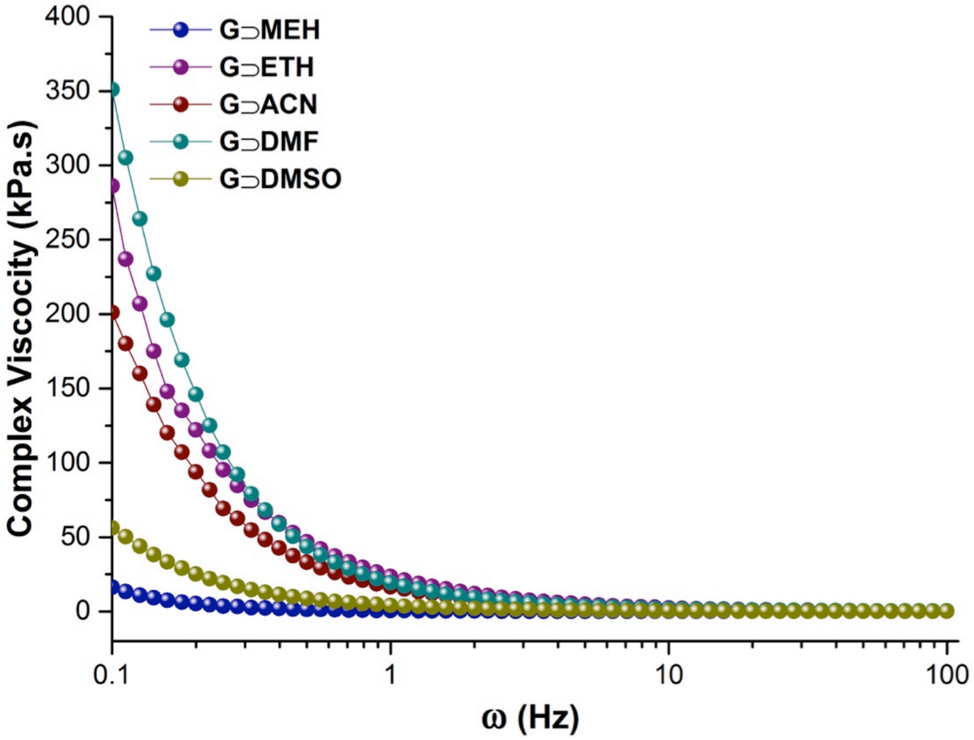

Figure S22. Viscosities of all gel samples measured against frequency, from 0.1 to 100 Hz.

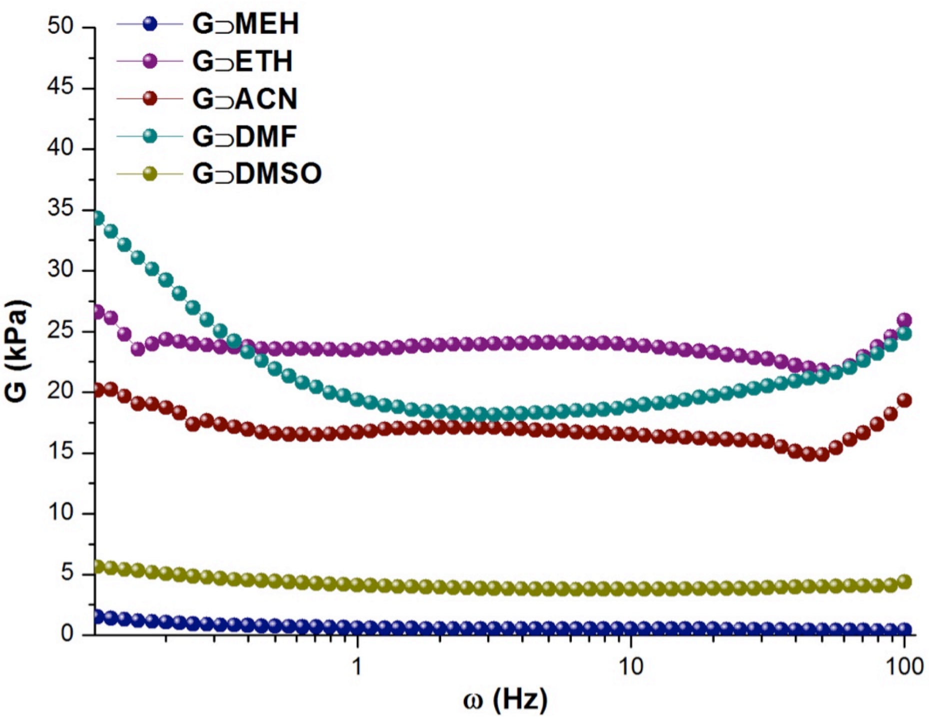

Figure S23. Shear modulus (G) of all gel samples, calculated from the storage (G') and loss (G") components of the moduli, where $|G| = \sqrt{(G')^2 + (G'')^2}$.

Thixotropic behavior of G⊃DMSO hybrid material

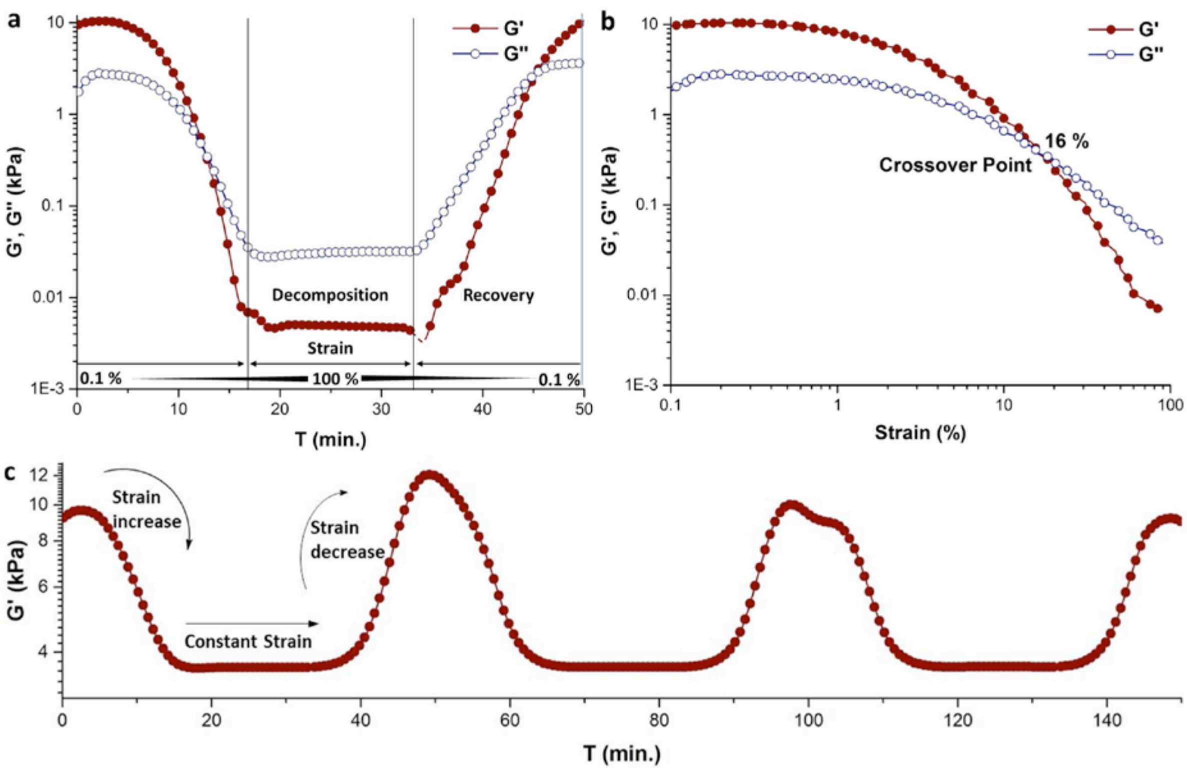

Figure S24. Dynamic strain sweep (DSS) measurement on stimuli-responsive **G⊃DMSO** to confirm its (a) thixotropic behavior, and (b) to determine the maximum strain tolerance limit (ω = 0.1 Hz). Gel phase recovery in 15 minutes is in agreement with the observed sol to gel transition time lapse (mentioned in the main manuscript i.e. 10-15 minutes) observed for mechanical stimulus (c) strain sweep measurement demonstrating the non-linear viscoelastic response of reversible gel phase for multiple consecutive strain cycles.

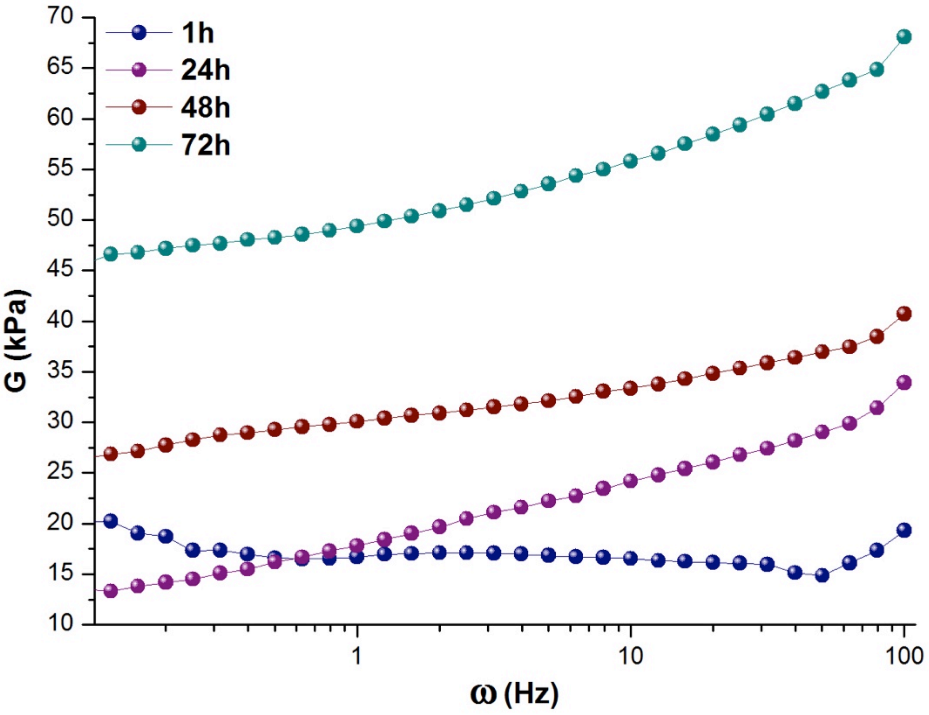

Figure S25. Shear modulus (G) of **VE⊃ACN** measured at different times, obtained from storage and loss moduli at corresponding time points of Fig.4(d),(e) in main manuscript

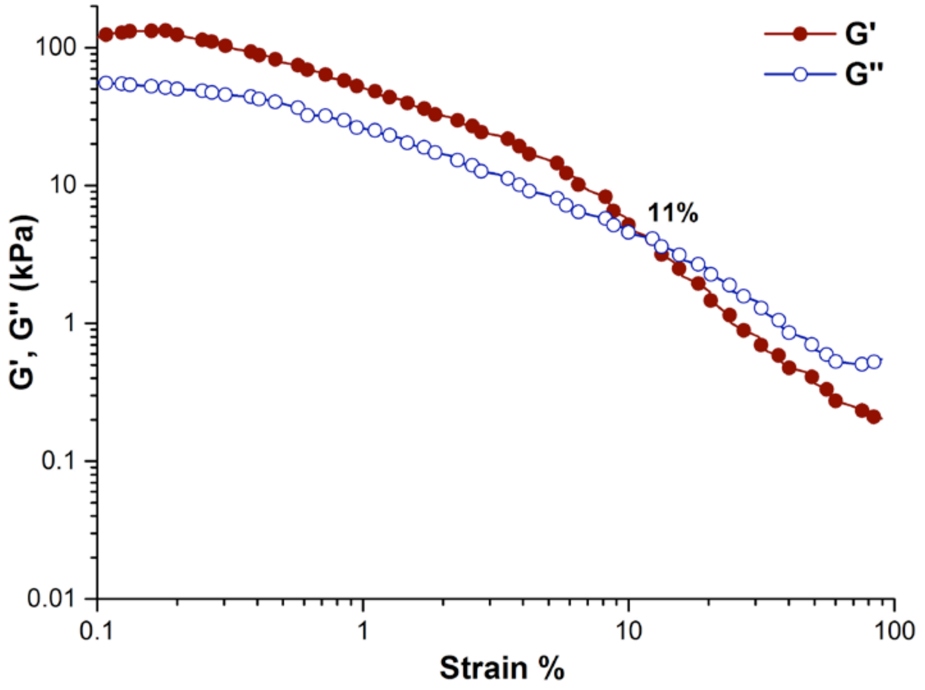

Figure S26. Dynamic strain sweep (DSS) measurement on **VE⊃ACN** to estimate the maximum strain tolerance of the compound (ω = 0.1 Hz).

Viscoelastic behavior – creep and stress relaxation

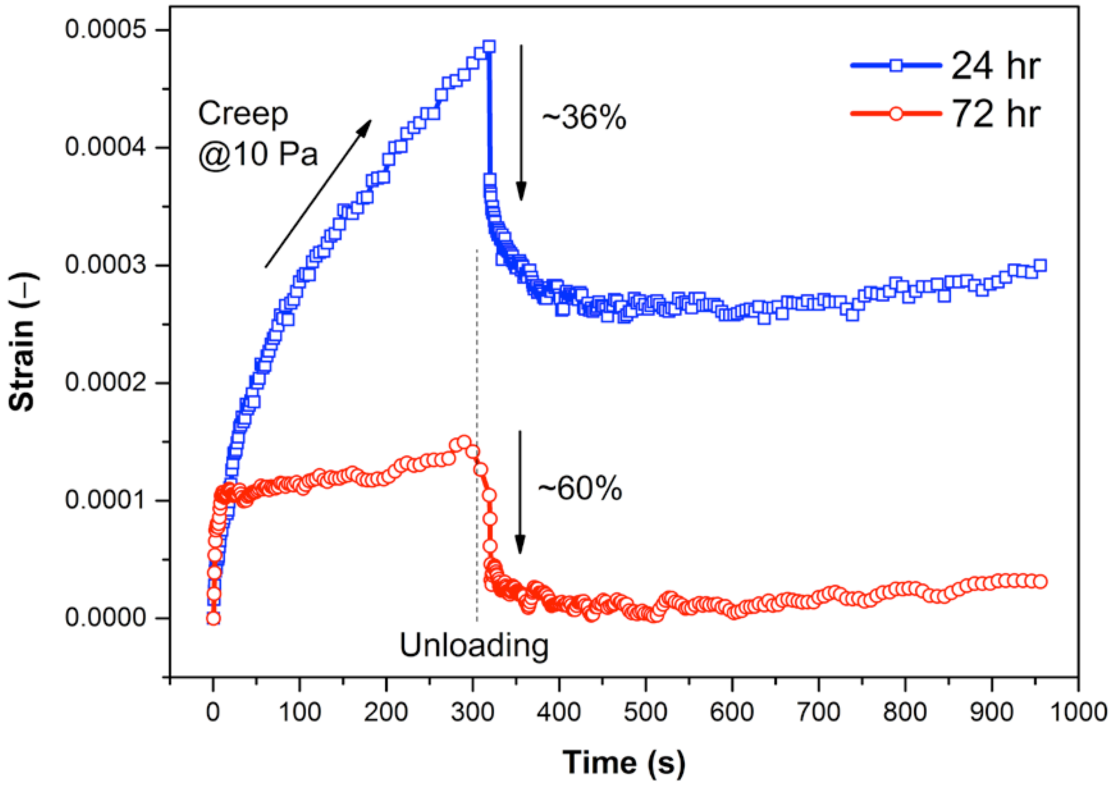

Figure S27. Creep-recovery tests at room tempeture performed under a constant shear stress τ = 10 Pa, and followed by complete unloading at 300 sec. The resulting strains as a function of time shows the visco-elastic nature of the **G⊃ACN → VE⊃ACN** compounds at 24 hr and 72 hr, with respectively experiencing ~36% and ~60% in strain recovery (w.r.t. maximum strain prior to unloading). This finding confirms that the transformation of 'weak' **G⊃ACN** gel network (suffers from substantial creep) to the **VE⊃ACN** semi-solid material confers major improvement in mechanical rigidity.

Electrical conductivity measurements

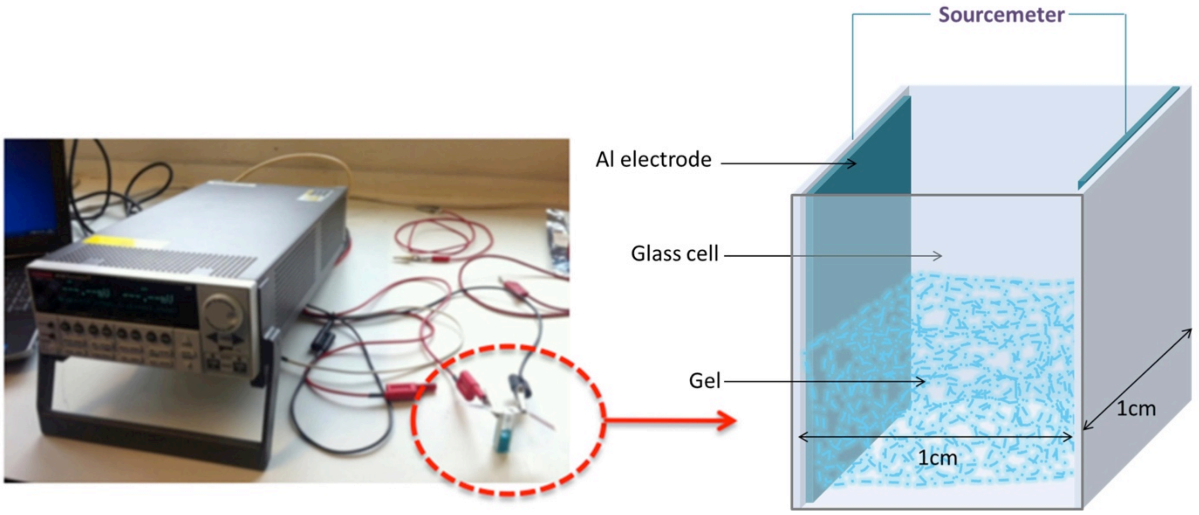

Figure S28. Experimental setup and geometry of test cell used in electrical conductivity measurements of gel samples

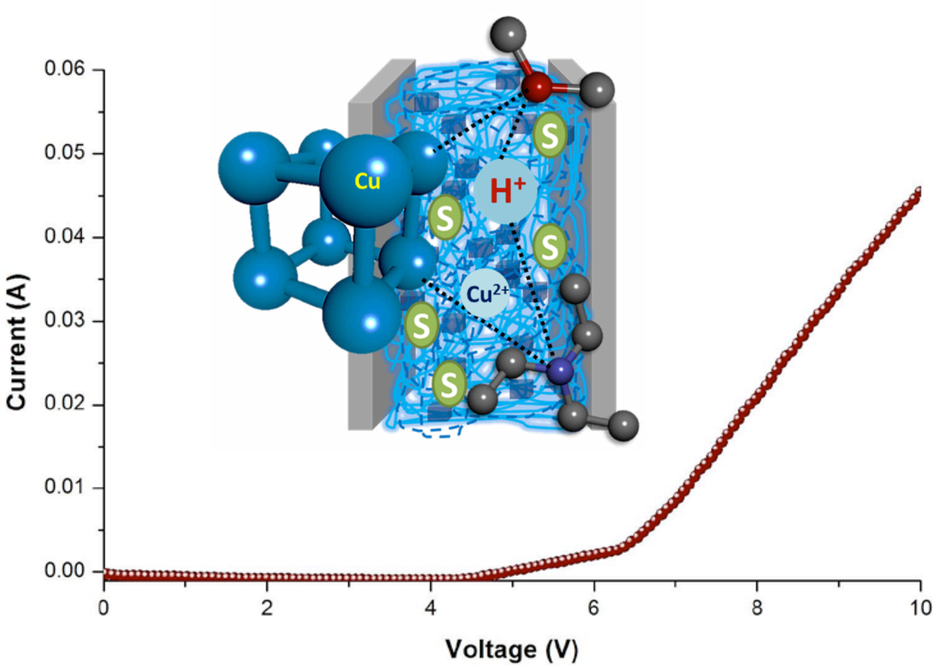

Figure S29. Current versus voltage (I-V) curve for the viscoelastic solid **VE⊃ACN.** Conductivity measurements were performed by pressing a thin layer of **VE⊃ACN** between two aluminum electrodes with dimensions of A = 42 mm^2 and L = 9 mm. Inset: illustration depicting potential charge carriers encompassing reactive Cu metal sites in MOF nanoparticles, weakly interacting Cu(II) cations and other ionic species present in fibrous network, in addition to H^+ ions from occluded solvent.

MOF thin films fabricated from sol-gel method

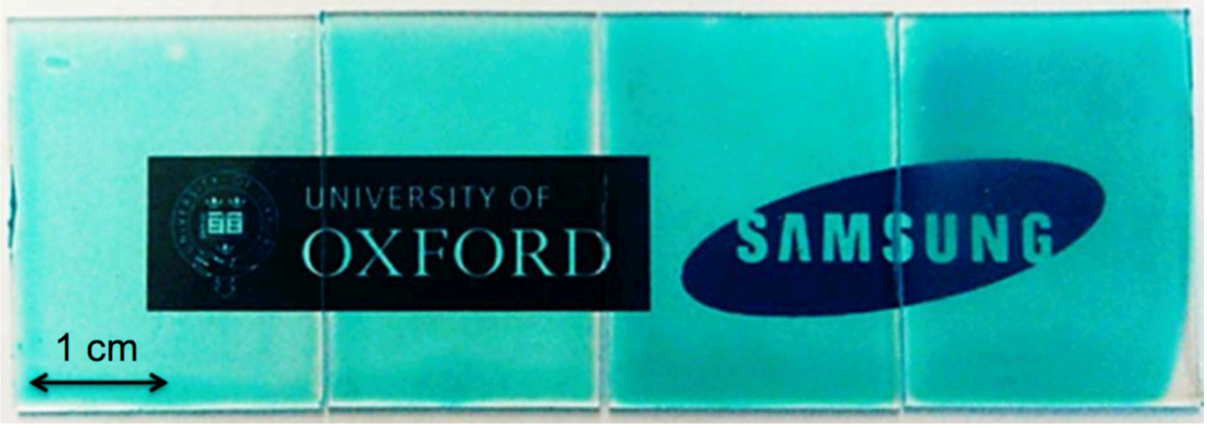

Figure S30. MOF thin films with increasing thickness, from left to right *ca*. 1, 2, 5 10 µm, obtained from sol-gel method of **G⊃DMSO** and deposited *via* doctor-blade technique. Film thicknesses were characterized by infinite focus microscopy using a 3D non-contact optical profilometer.

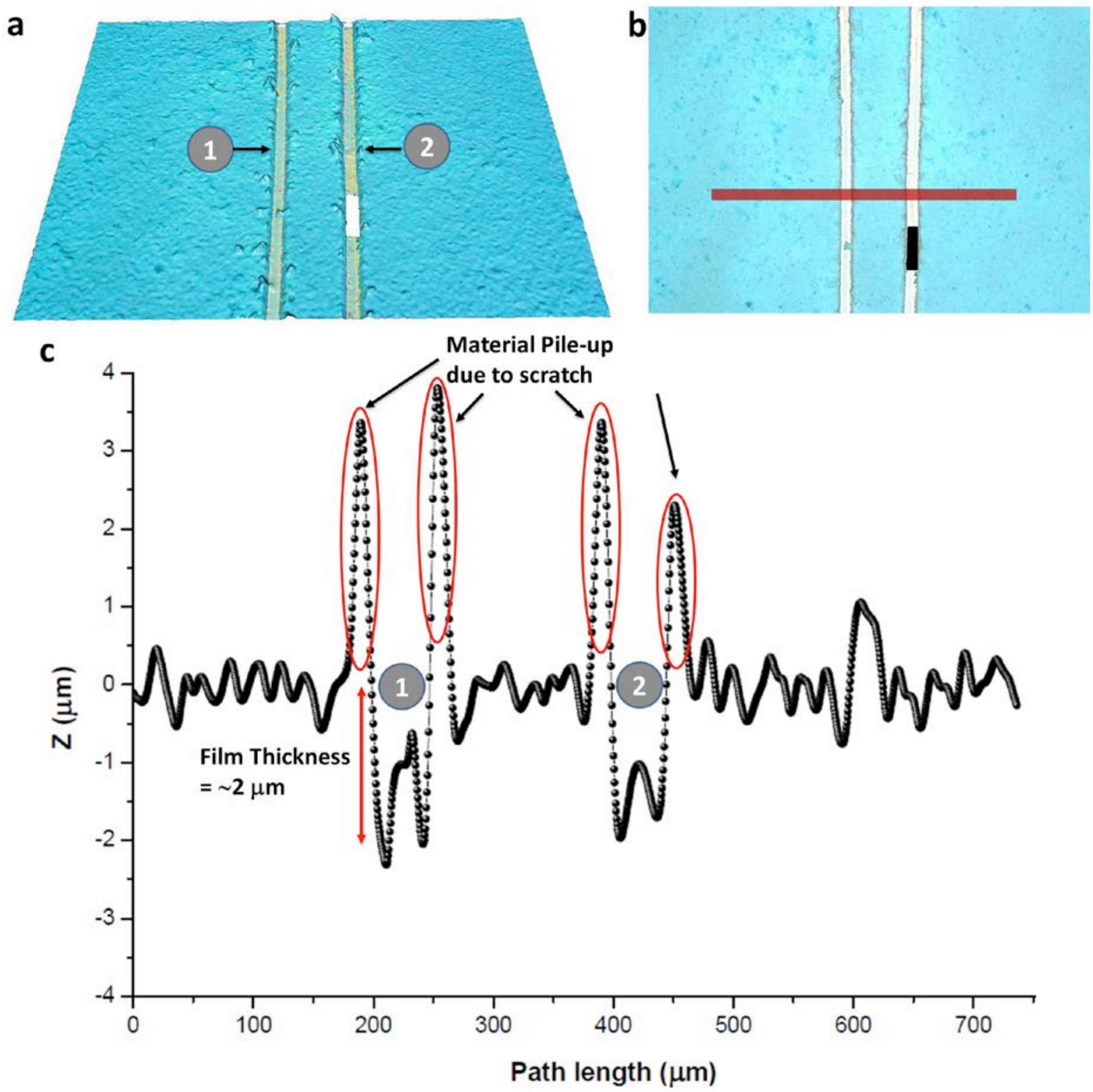

Figure S31. Example of thin film thickness measurement method involving optical imaging of the material through the scratches made on film surface: (a) 3D optical colour image determined *via* non-contact profilometer (Infinite Focus Alicona), and (b) 2D image of the sample with two different scratches imaged in a single scan showing (c) film thickness of ~2 µm. Film obtained from the sol of **G⊃MEH**.

References

1 Shoaee, M., Anderson, M. W. & Attfield, M. P. Crystal growth of the nanoporous metal-organic framework HKUST-1 revealed by in situ atomic force microscopy. *Angew Chem Int Ed Engl* **47**, 8525-8528, doi:10.1002/anie.200803460 (2008).

2 Jia-Min Chen, J.-J. S., Wei-Wei Huang, Yan-Ni Lao, Shi-Ping Yang. Triethylaminium-trimesate-trimesic acid-water (1/1/1/2). *Acta Crystallographica Section E: Structure Reports* **63**, o3053 (2007).

3 Davey, R. J., Brychczynska, M., Sadiq, G., Dent, G. & Pritchard, R. G. Crystallising trimesic acid from DMSO solutions - can crystallography teach us anything about the process of crystal nucleation? *Crystengcomm* **15**, 856-859, doi:10.1039/c2ce26712h (2013).
